# Laser synthesis of nanoparticles in organic solvents – products, reactions, and perspectives

**DOI:** 10.3762/bjnano.15.54

**Published:** 2024-06-05

**Authors:** Theo Fromme, Sven Reichenberger, Katharine M Tibbetts, Stephan Barcikowski

**Affiliations:** 1 Technical Chemistry I and Center for Nanointegration Duisburg-Essen (CENIDE), University of Duisburg-Essen, Universitätsstrasse 7, 45141 Essen, Germany; 2 Department of Chemistry, Virginia Commonwealth University, Richmond, VA 23284, USA

**Keywords:** alloy, photochemistry, pyrolysis, radicals, surface chemistry

## Abstract

Laser synthesis and processing of colloids (LSPC) is an established method for producing functional and durable nanomaterials and catalysts in virtually any liquid of choice. While the redox reactions during laser synthesis in water are fairly well understood, the corresponding reactions in organic liquids remain elusive, particularly because of the much greater complexity of carbon chemistry. To this end, this article first reviews the knowledge base of chemical reactions during LSPC and then deduces identifiable reaction pathways and mechanisms. This review also includes findings that are specific to the LSPC method variants laser ablation (LAL), fragmentation (LFL), melting (LML), and reduction (LRL) in organic liquids. A particular focus will be set on permanent gases, liquid hydrocarbons, and solid, carbonaceous species generated, including the formation of doped, compounded, and encapsulated nanoparticles. It will be shown how the choice of solvent, synthesis method, and laser parameters influence the nanostructure formation as well as the amount and chain length of the generated polyyne by-products. Finally, theoretical approaches to address the mechanisms of organic liquid decomposition and carbon shell formation are highlighted and discussed regarding current challenges and future perspectives of LSPC using organic liquids instead of water.

## Introduction

**Figure 1:**
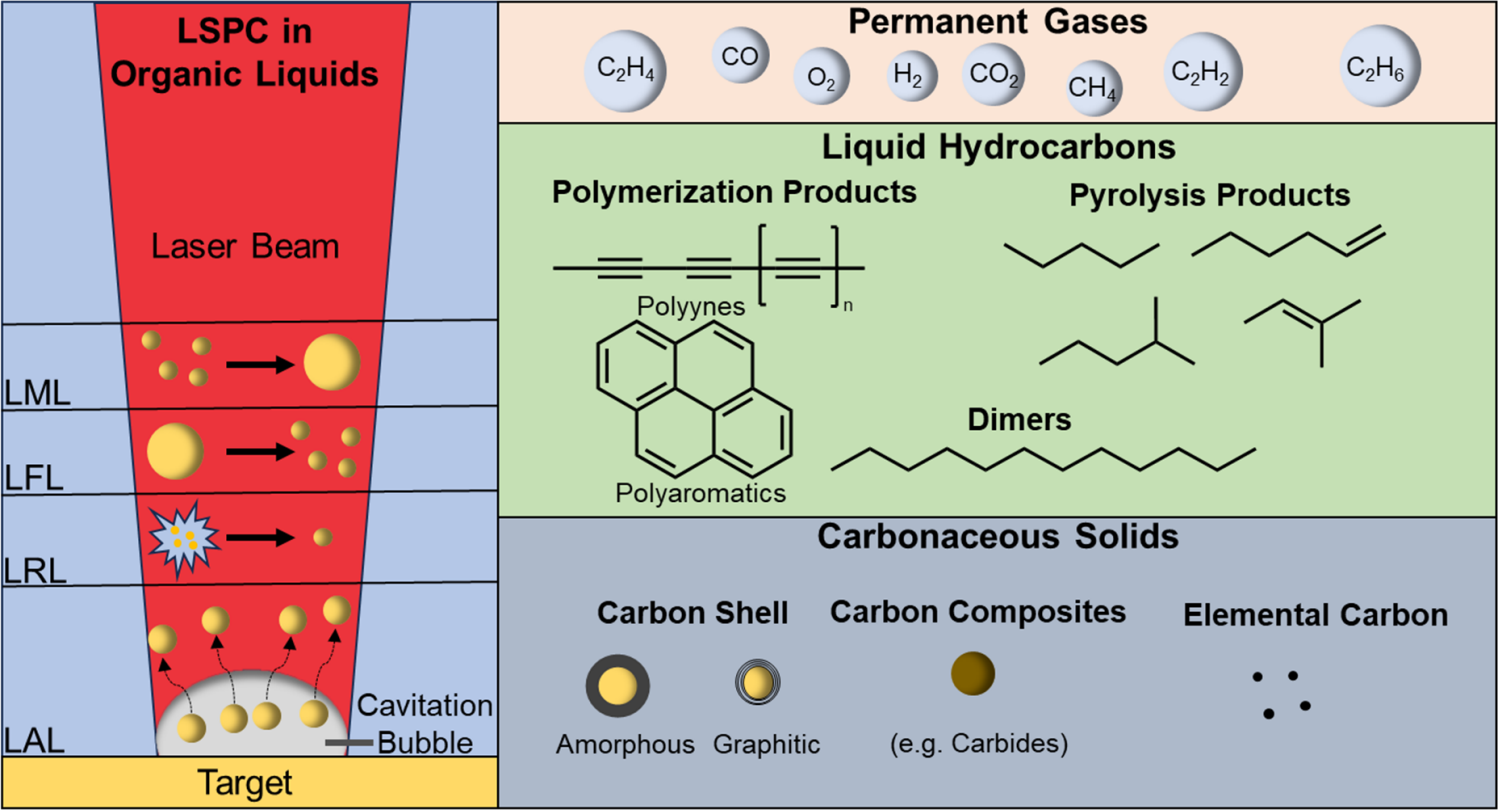
Variants of pulsed laser synthesis and processing in organic liquids as well as classification of the by-products including reported formation pathways. The LSPC variants can be classified by the laser fluence regime (typically, LAL > LRL > LFL > LML), the characteristic size of starting material (LAL > LML > LFL > LRL), or obtained particle sizes (typically, LML >> LAL > LFL ≈ LRL). The synthesis products and by-products are schemed on the right side and may be categorized into three classes: (i) gaseous by-products (i.e., H_2_, CO, CO_2_, and C_2_H*_x_*) emerging as persistent gas bubbles, (ii) liquid hydrocarbons resulting from chemical reactions (i.e., pyrolysis, polymerization, and dimerization), and (iii) carbonaceous solids consisting of amorphous carbon shells, graphitic onion-like carbon shells, elemental carbon, and carbon composites.

Since the first reports from Fojtik and Henglein on nanoparticle synthesis [1] and Patil et al. on reactive target modification [2], pulsed laser synthesis and processing of colloids (LSPC) has been shown to be a scalable [3–6] and versatile technique for nanoparticle synthesis, comprehensively reviewed regarding fundamentals and applications [7]. The colloids reported in the literature contain mainly inorganic particles (hence, they are the focus of this review), although the literature on organic particle synthesis has been reported as well, ranging from dyes [8–9] to natural substances [10–11] and drugs [12]. LSPC generation of inorganic particles is a physicochemical approach that claims to synthesize particles without surfactants or molecular additives. In contrast to other approaches of nanoparticle synthesis, LSPC only requires the neat (elemental) target materials while no other precursors or ligand exchange reactions are needed. Furthermore, properties such as surface structure or crystallinity can be tailored by adjusting experimental conditions while retaining the initial chemical composition of the educt material [13]. LSPC can be classified into the method variants of laser ablation in liquid (LAL), laser reduction in liquid (LRL), laser fragmentation in liquid (LFL), and laser melting in liquid (LML), which are schematically shown in Figure 1. Molecular precursors are only required in LRL, whereas the other variants employ a solid as starting material, which is ablated/fragmented/molten in the dispersing liquid. Hereby, in ideal cases that fulfill the “purity” claim, the liquid shall not be degraded into reaction products that may adsorb to the nanoparticle surface as they are difficult to remove afterward. Here, water is less critical than organic solvents, where liquid hydrocarbons and other species may be unintendedly created as by-products, potentially found as surface adsorbates on the produced colloidal particles, compromising the nominal purity. However, molecular surface adsorbates may contribute to enhanced functionality in application scenarios, for example, through hindering kinetic accessibility and, thereby, slowing down oxidation, providing conductivity via carbon shells, or providing steric stability against aggregation.

Regardless of the liquid medium, the LSPC variants have process-specific characteristics, mainly classified by the product but also by the starting material. LAL, LFL, and LRL yield nanoparticles as products, whereas LML creates submicrometer spheres. LAL, LFL, and LML process solids, whereas LRL employs solvates as precursors (Figure 1). In detail, LAL describes the laser irradiation of a macroscopic target and the subsequent removal of surface matter, which leads to the formation of nanoparticles and can be performed in either aqueous media [4–5,14] or organic solvents [14–18]. Further, it is possible to synthesize metastable phase nanomaterials (NMs) that are hardly obtainable by conventional, chemical methods [19–24]. LFL utilizes commercial-grade powders or nanoparticles to downsize the particles by laser irradiation with high fluences [7,25]; LML, in contrast, is used to isochorically alter the shape or increase the size of nanoparticles by low-fluence irradiation of nanoparticles [26–27]. A variant of LSPC is reactive laser ablation, fragmentation, or melting in liquids (RLAL, RLFL, or RLML), which refers to the synthesis of nanoparticles wherein molecular or galvanic replacement precursors, such as metal salts, are added to react in situ [7]. The added precursors take part in chemical reactions leading to the generation of products that differ from the initial target’s composition [28–32]. Besides LAL, LFL, and LML, where a solid bulk or particulate material is laser-excited, another method to synthesize colloidal nanoparticles is the laser reduction in liquid (LRL), where the liquid contains solvated molecular precursors and is excited itself. LRL was first published by Shafeev et al. in 1986, who reduced triphenylphosphine Au(I) complexes to form Au nanoparticles on different materials such as GaAs [33] and was recently extended to synchronous LRL of multiple elements into high-entropy material nanoparticles formed in-place on various substrates [34]. In general, LRL utilizes metal salt or metal-organic complex solutions to form nanoparticles by photochemical reduction of the respective metal ions [35–39]. Besides the formation of metal nanoparticles, LRL can also induce nuclear reactions such as the (alpha) decay of uranium in the proximity of Au nanoparticles [40–41]. This LSPC process variant has also been called pulsed laser photoreduction/-oxidation in liquids (LPL) [42], and LRL has recently been reviewed by the Tibbetts group emphasizing the involved redox reactions and radical species [43]. Besides the reactions involving metal salts during LRL, gas formation [44–47] and solvent decomposition [48–51] have also been reported, highlighting the importance of chemical reactions during the processes, although LSPC is often considered to be a physical synthesis method. Moreover, the irradiation of pure organic solvents with femtosecond or picosecond radiation led to the formation of numerous products induced by the intense conditions, which can be attributed to laser-induced optical breakdown and/or shockwaves. The optical breakdown of the solvent and shockwaves can initiate bimolecular reactions that primarily lead to dimerization but also allow for fragmentation, polymerization, and other reactions to occur [52]. Figure 1 schematically summarizes the classification of LSPC methods and the generated particles (left side), and by-products obtained during the processes by solvent decomposition (right side) in organic liquids. For the particle formation mechanisms of these process variants, we refer to a recent LSPC review article [53]. While the nanoparticles obtained in aqueous liquids are at least partially oxidized [53–54], the conditions during LSPC in organic liquids are quite different. The solvent molecules themselves as well as the created hydrocarbons can adsorb on the nanoparticle surface and act as ligands. If a carbon shell is formed, it can be amorphous or onion-like graphitic. In addition, composites such as carbides, metallic glasses, or intermetallics can be synthesized. The origin of the formed carbon can be traced back to chemical reactions during the LSPC, which have been reported to be pyrolysis, di-/trimerization, and polymerization. The by-products of these reactions can be either liquid hydrocarbons, for example, polyynes or alkanes, or permanent gases forming persistent gas microbubbles.

There have been various reviews regarding nanoparticle synthesis (mainly addressing findings reported for aqueous media) [7,54–56], fundamental physical processes during LSPC [57–59], and the potential use of the laser-generated nanoparticles in nanomedicine [60–61] and catalysis [13,54,62–63], including electro- and photocatalysis [13]. The liquid’s influence on the nanoparticle properties as well as its decomposition products, in contrast, have received significantly less attention. Some past reviews addressed at least in part organic solvents as LSPC media. Batista et al. recently reviewed the possibilities of reactive laser synthesis [43]. While the chemical reactions occurring during reactive laser synthesis in aqueous solutions and organic solvents are discussed, the review highlights RLAL, RLFL, RLML, and LRL of various (base, noble, and alloy) metals and their possible use in the future. Marabotti et al., in contrast, focused on polyyne formation only, highlighting the different formation mechanisms, the effect of aqueous or organic solvents, the influence of the irradiated target, and nanocomposites based on sp-hybridized carbon chains [64]. Liang et al. specified the conditions for inhibition of phase crystallization and, hence, the formation of metallic glass nanoparticles in organic solvents, which was attributed to the carbon doping of the amorphous phase as well as carbon shell formation stabilizing the nanoparticles’ glass structure [65]. While these reviews consider at least in part the chemical reactions occurring in organic liquids, they focus on specific topics. Thus, this perspective article aims to survey the current state of knowledge on the laser-based synthesis of colloidal nanoparticles in organic solvents as well as the underlying chemical reactions and their influence on particulate properties. Additionally, the knowledge base on the role of volatile and non-volatile molecular products forming during LSPC will be addressed.

## Review

### LSPC in aqueous liquids

Laser-based nanoparticle synthesis in water is always accompanied by the production of gases [66–67]. Although gas formation has often been attributed to the vaporization of water, the formation of hydrogen and oxygen also occurs. Additionally, the formation of hydrogen peroxide was observed during LAL [50–51] and LRL [36]. Depending on the process, gas formation can be attributed to different redox reactions that contribute to nanoparticle formation. For the laser ablation, fragmentation, and melting processes, the nanoparticles are found to be at least partially oxidized, ranging from a surface oxidation of 5–10% for gold [68–69] to a completely oxidized bulk volume, for example, for nickel [70]. In contrast, laser reduction in water leads to the reduction of metal salts and, thus, to the nucleation of metallic nanoparticles [36,71]. Hence, water acts as an oxidizing agent in the context of LAL, LFL, and LML, while it is a reducing agent during LRL, indicating an ambivalent behavior during the chemical processes. Recently, this ambivalent character has been demonstrated for iridium-based microparticle LFL, where elemental iridium as starting material was oxidized, and the oxidized iridium reactant was (partially) reduced, under identical processing conditions with identical product nanoparticle diameters of 3 nm [72]. Moreover, the degree of reduction or oxidation was gradually tuned to reach a thermodynamically driven equilibrium with the cumulative laser pulse energy input [72–73]. It indicates a certain controllability of the redox reactions occurring during reductive or oxidative laser processing in aqueous media at constant particle size. Furthermore, focusing laser pulses in CO_2_-saturated water leads to the reduction of CO_2_, which selectively yields CO [74] or oxocarbon-encapsulated nanoparticles [75–76]. Beyond the formation of carbon monoxide, the direct reduction of permanent solutes in liquids via LRL, sometimes termed laser bubbling in liquid (LBL), has been demonstrated recently for alternative reactants, such as hydrogen extraction from ammonia [77] or methanol [78], and direct synthesis of HCN [79].

The oxidation and phase change of the target surface during LAL was initially published by Ogale et al. [80] in 1987, and nanoparticle oxidation has been addressed in the literature frequently afterwards [53–54,68–70]. During the plasma and cavitation bubble phase, reactive oxygen species (ROS), for example, hydrogen peroxide, hydroxyl radicals, or dissolved oxygen, react with the particles leading to their surface oxidation. During irradiation of water with intense laser pulses, a weakly ionized plasma forms because of optical breakdown, supercontinuum emission, or both. Optical breakdown occurs when the free-electron density surpasses a critical value, resulting in a high-density plasma, and the optical breakdown threshold is significantly reduced in the presence of metal nanoparticles [49,81–82]. Supercontinuum emission can occur at low fluences, when pulses shorter than 100 fs are used, and will produce a low-density plasma [83]. More recently, the Vogel group studied the energy spectrum of laser-induced conduction band electrons in water by introducing a simplified splitting scheme and corresponding rate equations, well suited also for the calculation of energy spectra at long pulse durations and high irradiance [84]. This approach provides the essential understanding of the dependence of electron energy spectra on laser pulse duration, wavelength, and irradiance, which opens pathways for inducing energy-specific molecular modifications in dielectric media, such as water and even aqueous solutes. Thus, this model formed the basis that enabled the derivation of yield functions for a variety of direct electron-mediated DNA damage pathways and indirect damage by •OH radicals resulting from laser and electron interactions with water [85]. In general, LSPC in aqueous solutions produces free electrons and ROS. The ROS lead to a certain degree of nanoparticle (surface) oxidation depending on the material’s standard electrochemical reduction potential for LAL and LFL. For LRL, both electrons and ROS contribute to the reduction reactions of the metal salts resulting in nanoparticles, depicting the ambivalent behavior of ROS.

**Figure 2:**
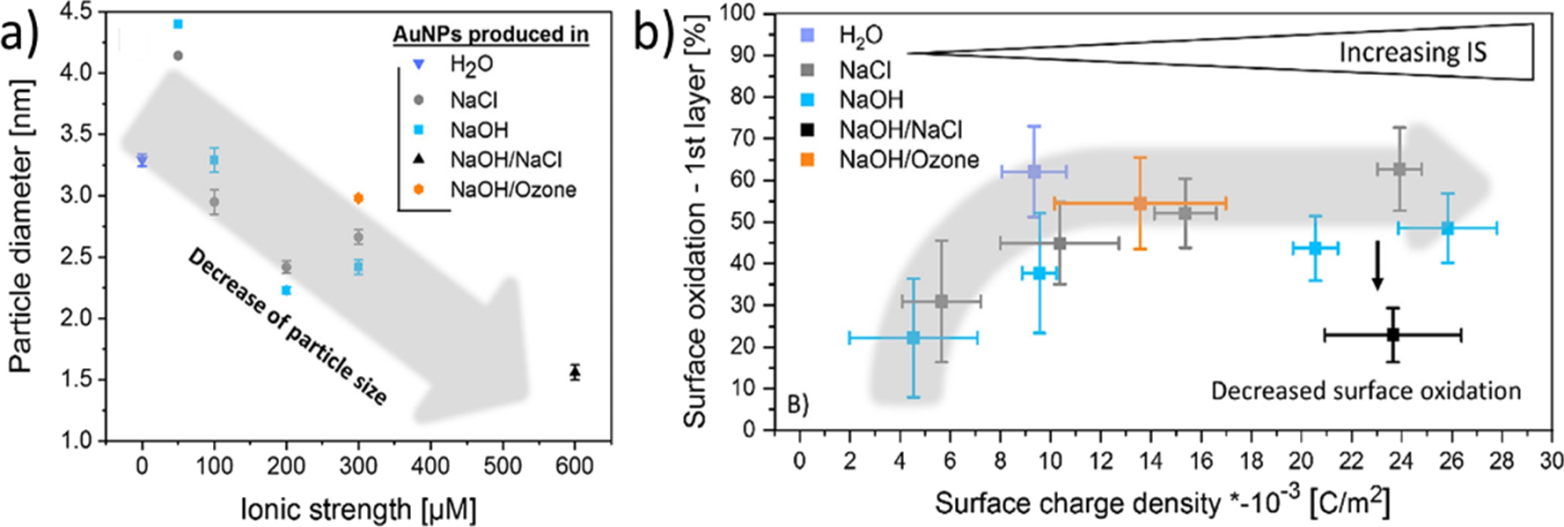
Anion solute effects on pulsed laser synthesis in water. (a) Effect of ionic strength on the gained particle diameter during the LFL of Au. (b) Relation between surface oxidation of the first atomic layer and surface charge density delivered by adsorbed anions. Figure 2 was reprinted with permission from [88], Copyright 2020 American Chemical Society. This content is not subject to CC BY 4.0.

Nanoparticle surface oxidation during LSPC can either be suppressed by degassing the water used [86–87] or amplified by adding salts such as NaCl [88]. Ziefuß et al. found an inverse linear trend during the LFL of gold when correlating the ionic strength of the added salts and the obtained gold particle size (Figure 2). The surface oxidation increased with the anion’s surface charge density until a plateau of around 60% was reached. A further increase in ionic strength showed no change in surface oxidation, which was attributed to an accumulation of anions in the Helmholtz layer indicated by zeta potential measurements. Scaramuzza et al., in contrast, investigated non-ionic additives and their influence on structure and composition during the ablation of metastable AuFe alloys [20]. They used ethanol and water as solvents and added different additives. In the case of gaseous additives (nitrogen, carbon dioxide, and argon), they saturated the solution by bubbling. Further additives were 0.3 vol % hydrogen peroxide and 0.2 vol % water (for ethanol only). Depending on the chosen additive, they found a different degree of oxidation of the alloy nanoparticles. The highest oxidation was found for the mixture of water and hydrogen peroxide, yielding AuFe nanoparticles with an oxide shell; hence, the authors declared the mixture as highly oxidizing. In contrast, ethanol and hydrogen peroxide brought forth AuFe nanoparticles without an oxide shell. A moderate oxidation was found for the ethanol–water mixture, leading to oxide crescents on the nanoparticle surface; nitrogen-saturated ethanol led to scarcely oxidized AuFe nanoparticles. However, it has to be noted that the addition of such agents influences the ablation efficiency and gas formation. Scaramuzza et al. found varying ablation rates and cavitation bubble sizes depending on the used additive–solvent combination [20], and Zhang et al. found higher yields of gases when working in ethanol–water mixtures [44]. This enhanced gas production can be used to alter the structure of the generated nanoparticles. Laser ablation in water–ethanol mixtures was reported to yield an increased amount of hollow nanoparticles compared to pure water, which was mainly attributed to an elongated lifetime of the cavitation bubble in the mixture [89–90]. It is further possible to form porous nanoparticles when the liquid is saturated with hydrogen, depending on the specific metals and their properties (e.g., hydrogen permeability and diffusion coefficient of hydrogen) [91]. To summarize, the generated nanoparticles can be oxidized or reduced depending on process parameters such as gaseous additives or atmospheric conditions, liquid additives, or salts, which determine the degree of oxidation, nanoparticle structure, productivity, and gas formation rates.

**Figure 3:**
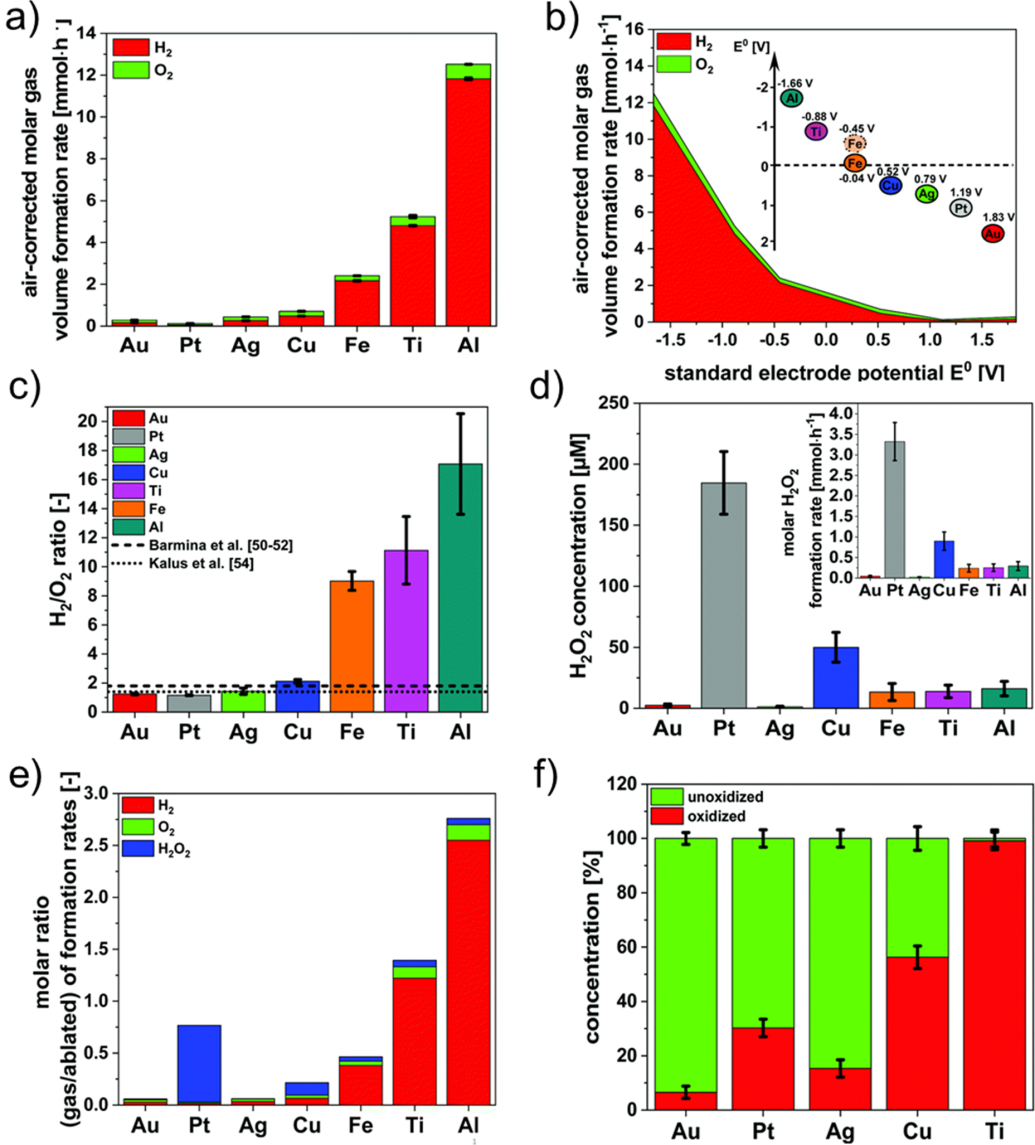
Water splitting and gas formation during LAL of seven metals with increased standard electrochemical reduction potential. (a) Molar gas volume formation rate obtained by LAL of the respective metals in water. (b) Molar hydrogen and oxygen gas volume formation rates depend on the standard electrochemical reduction potential of the used metals. (c) H_2_/O_2_ ratio gained during the LAL of metals compared to values known from literature. (d) H_2_O_2_ concentrations as well as the molar H_2_O_2_ formation rate (insert) found after laser ablation. (e) Molar ratio of formed by-products related to the molar nanoparticle productivity during the LAL in water. (f) Oxidation degree of ablated metal nanoparticles as measured by XPS. Figure 3 was republished with permission of PCCP Owner Societies, from [50–51] (“Determining the role of redox-active materials during laser-induced water decomposition” by M.-R. Kalus et al., *Phys. Chem. Chem. Phys.*, vol. 21, issue 34, © 2019 and the related correction); permission conveyed through Copyright Clearance Center, Inc. This content is not subject to CC BY 4.0.

Further differences have been found depending on the ablated metal. Gold, for instance, shows surface oxidation degrees of 5–10% [68–69], while platinum surfaces are partially oxidized with 20–73% [70,92], and nickel particles are completely oxidized [70]. This was further investigated by Kalus et al., who ablated seven different metals (Au, Pt, Ag, Cu, Fe, Ti, and Al) in water while quantifying the formed hydrogen and oxygen via the liquid displacement method and gas chromatography (Figure 3) [51]. The amount of formed hydrogen was found to be inversely correlated with the metal’s standard electrochemical reduction potential, resulting in higher hydrogen formation rates during the ablation of less noble metals such as Ti, Fe, and Al. As the H_2_/O_2_ ratio follows the same trend, redox reactions occurring between the formed nanoparticles and the formed oxygen species lead to the particles’ surface oxidation. This correlation shows that metals with a lower standard electrochemical reduction potential than hydrogen (*E*^0^ < 0 V) undergo bulk oxidation during the ablation process, while metals with a higher standard electrochemical reduction potential are less oxidized. In addition to hydrogen and oxygen, hydrogen peroxide was also formed during the ablation process. The formation rate of hydrogen peroxide during LAL of Pt (and Cu) was superior compared to that of the other metals (Figure 3d,e) because of surface-catalytic reactions of the metal [51]. In summary, the total water decomposition products formed during ablation increase for less noble metals, which in turn form higher fractions of surface oxide because of the redox reactions of the released oxygen with the generated nanomaterials, resulting in a depletion of oxygen and a higher fraction of the generated hydrogen.

**Figure 4:**
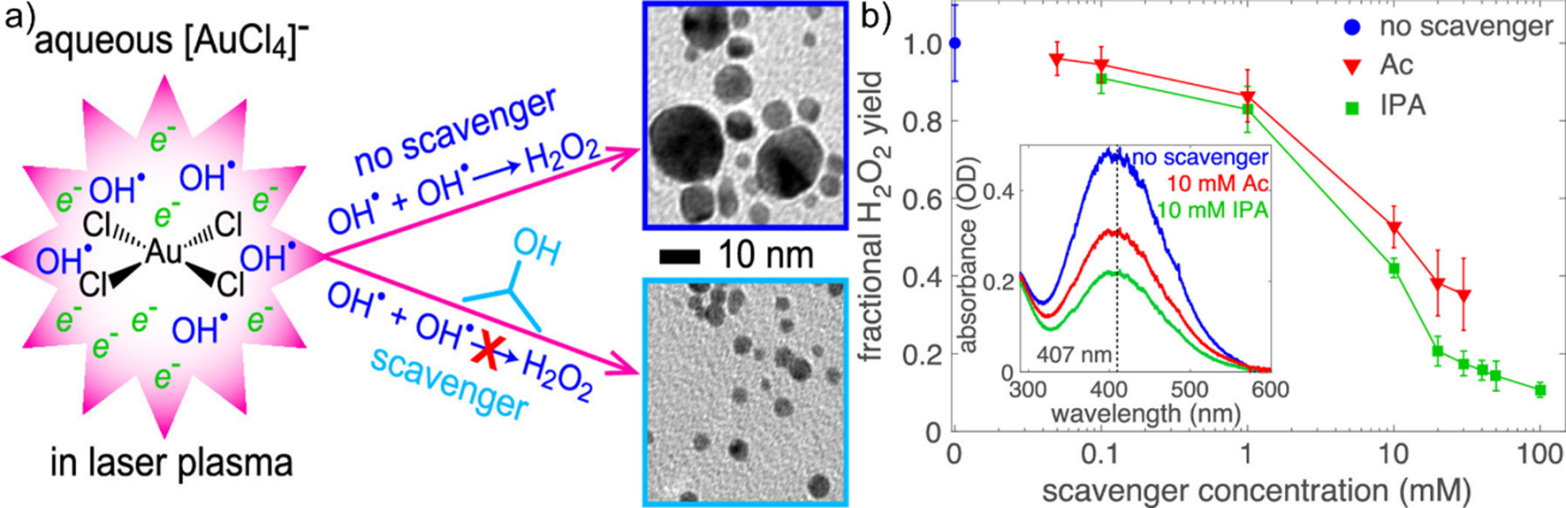
(a) Influence of hydroxyl radical scavengers on the size distribution obtained during the LRL of AuCl_4_^−^ in water. (b) Quantified yields of H_2_O_2_ generated in aqueous scavenger solutions containing varying amounts of 2-propanol and acetate. Inset: absorption spectra of pertitanic acid (TiO_2_H_2_O_2_) in water and radical scavenger solutions. Figure 4 was reprinted with permission from [48], Copyright 2019 American Chemical Society. This content is not subject to CC BY 4.0.

The process of LRL stands in direct contrast to oxidation during LAL and LFL, but the (effective) reducing character of the water can also be explained by the previously mentioned ROS, solvated electrons, hydrogen radicals, and hydroxyl radicals. Whereas solvated electrons are generally accepted as the dominant reducing agents in LRL, the hydrogen peroxide produced by the recombination of hydroxyl radicals significantly contributes to the reduction of Au salts during LRL. Moreover, formed molecular hydrogen can contribute to reduction. Hydrogen formation was first reported by Maatz et al. in 2000 by irradiating water, saline solutions, and gelatine phantoms [66]. Meader et al. correlated the formation of free electrons and H_2_O_2_ with the [AuCl_4_]^−^ reduction rate through a two-step autocatalytic nanoparticle growth mechanism [71]. The autocatalytic growth step is driven by H_2_O_2_ and can be slowed down by adding hydroxyl radical scavengers resulting in smaller nanoparticles (Figure 4) [48]. Hence, the water decomposition leads to the formation of reducing agents for metal nanoparticle formation during irradiation of metal salt solutions with ultrashort pulses. Several LRL syntheses of nanoparticles, which provide either noble metals, base metals, or oxides, are available and, given the recent review of Frias Batista et al., will not be listed here [43].

Overall, LSPC in aqueous liquids is characterized by in situ water splitting. More precisely, highly reactive radicals and free electrons are formed, which act as reducing agents for metal precursor salts during LRL, as well as molecular hydrogen, oxygen, and hydrogen peroxide. However, when LAL or LFL of metals is performed, the targets are oxidized by redox reactions with these ROS. The degree of target oxidation and hydrogen formation rate are inversely correlated and determined by the metal’s standard electrochemical reduction potential. The relation of such material-related effects during LAL would require ideally more single-pulse studies [93] as, during prolonged LAL, the formed nanoparticles will cause in-process LFL and LML, making it difficult to distinguish between reaction in the liquid caused by the target ablation and nanoparticle excitation. With increasing nanoparticle mass concentration, the fraction of permanent gas caused by nanoparticle excitation during LAL reaches already 50% of the total gas at 200 mg/L and can be responsible for as much as 80% of the gaseous laser synthesis reaction products [49].

### LSPC in organic solvents with and without solutes

While LSPC in aqueous solutions leads to (partial) oxidation of the generated nanoparticles in the colloid [50–51,94–96], the laser-based synthesis of nanoparticles in organic solvents is more likely to retain the initial composition of the material [86,97–98] or to form metal carbides [99–103] while modifying the surface with an amorphous or graphitic carbon shell [99,101,104–105]. As previously mentioned, gas formation is apparent during LSPC in organic solvents, too, but to a larger degree [44,106]. Kalus et al. found that LAL yielded around 20 times more gas in acetone and ethylene glycol than in water, reaching absolute values of 60 cm^3^/h (at only 5 W laser power), and specific values of 0.2 cm^3^/(mg·W) normalized to the ablated target mass and laser power [47]. This indicates the relevance of processes and by-products formed during LSPC in organic solvents as the gas formation rates are proportional to the applied laser power [107], resulting in the generation of large gas volumes when working with high laser powers, such as state-of-the-art pulsed lasers with several hundred of watts of output power. While the decomposition species of nanoparticle syntheses in water are limited, the possible products formed during irradiation in organic solvents are plenty. First, the ionization of the organic solvent molecules produces free electrons and radical cations [108–110] with longer lifetimes than in water [111]. Because of their longevity, the radical cations can participate in various reactions such as dehydration, dimerization, and hydrogen transfer before recombination [112–114]. This was recently shown for fs-laser irradiation of C_5_ to C_11_ alkanes by Ishikawa et al., who reported C–C bond formation. They analyzed the formed products via gas chromatography and found dimers with different constitutions to be the main products. Besides dimer formation, the production of shorter carbon chains (down to C_4_ for the irradiation of undecane), longer carbon chains (C*_n_*_+1_ to C_2_*_n_*_−1_), and, although in very small quantities, trimers were observed. They concluded that laser-driven mechanical shockwaves induced these bond-formation reactions [52]. Irradiation of benzene was shown to produce biphenyl as a dimerization product and higher molecular aromatics [115–116]. However, additional products including hydrogen, methane, acetylene, ethylene [116–117], and polyynes [117–118] were observed from ablation of benzene and toluene. Overall, a large quantity of different products was found for the irradiation of solvents with pulsed lasers. Although the reaction products resulting from the photolysis of solvents have been characterized reasonably well, the influence of nanoparticle production on photolysis reactions and by-product formation is largely unknown. Thus, this chapter will discuss the known by-products obtained during laser-based synthesis in organic solvents. Because of the manifold reaction products, the formation of gases will be discussed first, followed by hydrocarbons and carbonaceous products.

#### Permanent gas evolution during LSPC

**Figure 5:**
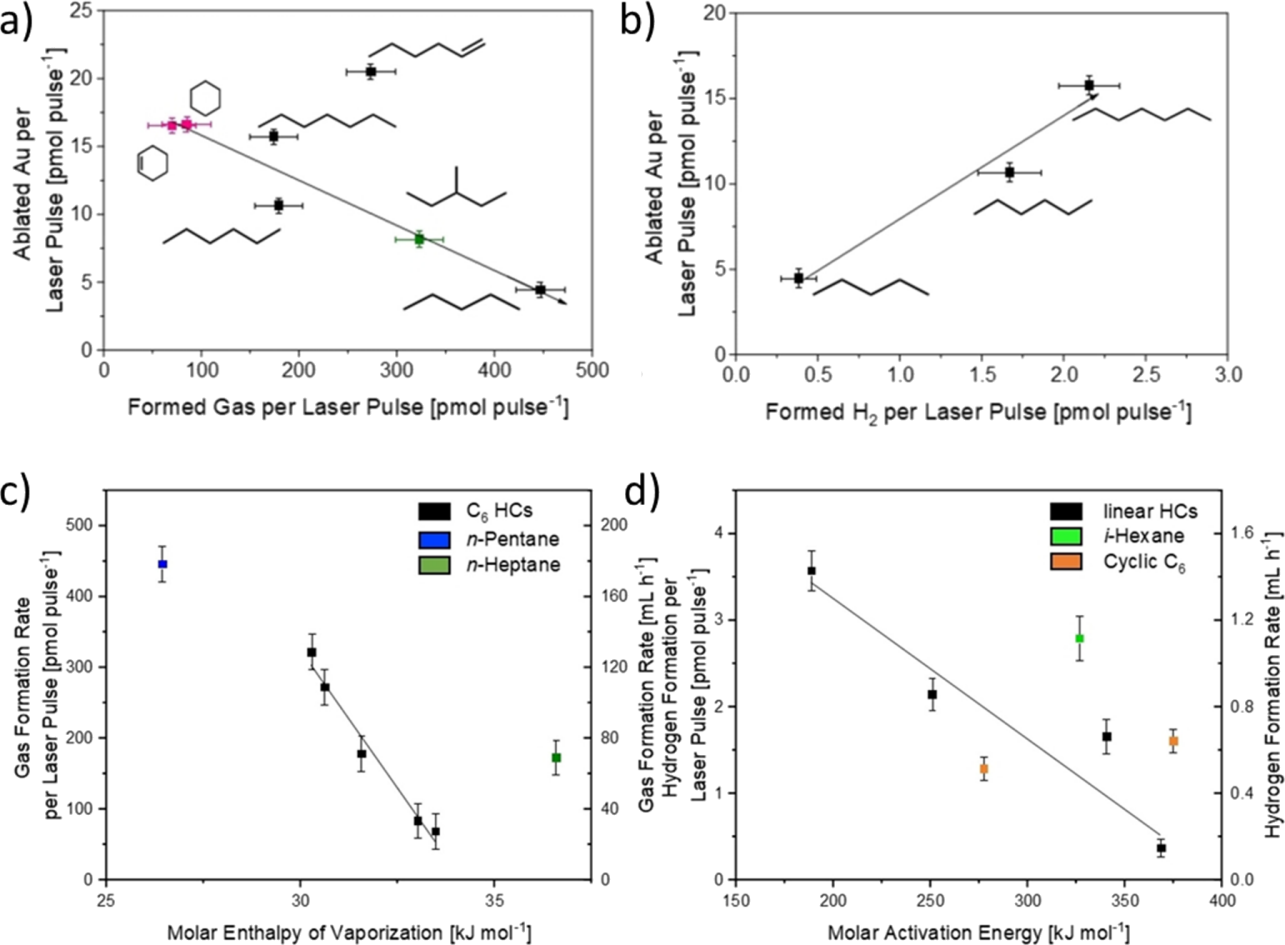
(a) Gas formation rates per laser pulse versus ablated gold per laser pulse. (b) Hydrogen formation rate per laser pulse versus ablated gold per laser pulse for linear hydrocarbons. (c) Influence of the molar enthalpy of vaporization of organic solvents on the gas formation rate during LAL of Au. (d) Impact of the molar activation energy during the first step in pyrolysis on the hydrogen formation rate during LAL. Figure 5 was reproduced from [107] (© 2023 T. Fromme et al., published by Wiley, distributed under the terms of the Creative Commons Attribution-NonCommercial 4.0 International License, https://creativecommons.org/licenses/by-nc/4.0/). This content is not subject to CC BY 4.0.

Compared to water, the laser irradiation of organic solvents (in the absence of nanoparticles) leads to decomposition reactions that form permanent gases. Baymler et al. irradiated water, ethanol, isopropyl alcohol, diethyl ether, and isobutanol with nanosecond laser pulses while quantifying the formed hydrogen pressure with amperometric sensors. The organic alcohols, while having a lower number of hydrogen atoms per molecule, showed a ten times higher hydrogen evolution rate than water [45]. Additionally, the molecular structure of the solvent affects the hydrogen evolution rate. Ethanol and isopropyl alcohol produced greater hydrogen yields than isobutyl alcohol and diethyl ether, which was attributed to the higher ratio of hydrogen to carbon and, thus, more C–H bonds relative to C–C bonds. In addition to hydrogen, other gases including methane, ethylene, acetylene, and ethane have been reported by multiple groups during irradiation of neat alkanes, alcohols, and aromatics with nanosecond, picosecond, and femtosecond laser pulses [116–117,119]. Gas formation also occurs during laser-based nanoparticle synthesis. Kalus et al. used the liquid displacement method to quantify the formed gases during the ablation of gold and observed gas evolution rates an order of magnitude (around 20 times) higher than in water [47]. Although hydrogen formation has been observed in a variety of ways [67,116,119–120], the influence of the solvent on the rate of hydrogen formation has not been discussed widely. Fromme et al. recently quantified the overall gas generation as well as hydrogen formation rate during the ablation of gold in several C_6_ solvents as well as *n*-pentane and *n*-heptane (Figure 5). While the overall gas formation was ruled by the solvent’s molar enthalpy of vaporization, the correlation of hydrogen formation with the physical properties of the solvents was not possible. Instead, the hydrogen formation depends on the liquid’s pyrolysis behavior. As such, 1-hexene and benzene, both prone to polymerization reactions and coke formation, showed the largest hydrogen production, while cyclohexane and cyclohexene released the lowest amount of hydrogen compared to other C_6_ solvents. Interestingly, ablation in *n*-pentane produced the least amount of hydrogen of all solvents. This may be attributed to differences in bond dissociation energies, which increase with decreasing solvent chain length as well as chemical constitution. Hence, the solvent decomposition and (permanent) gas evolution are influenced by both physical and chemical properties of the solvents. Interestingly, solvent decomposition could be connected to the chemical behavior of the solvent during pyrolysis, although laser ablation is not a thermodynamically driven process [107].

**Figure 6:**
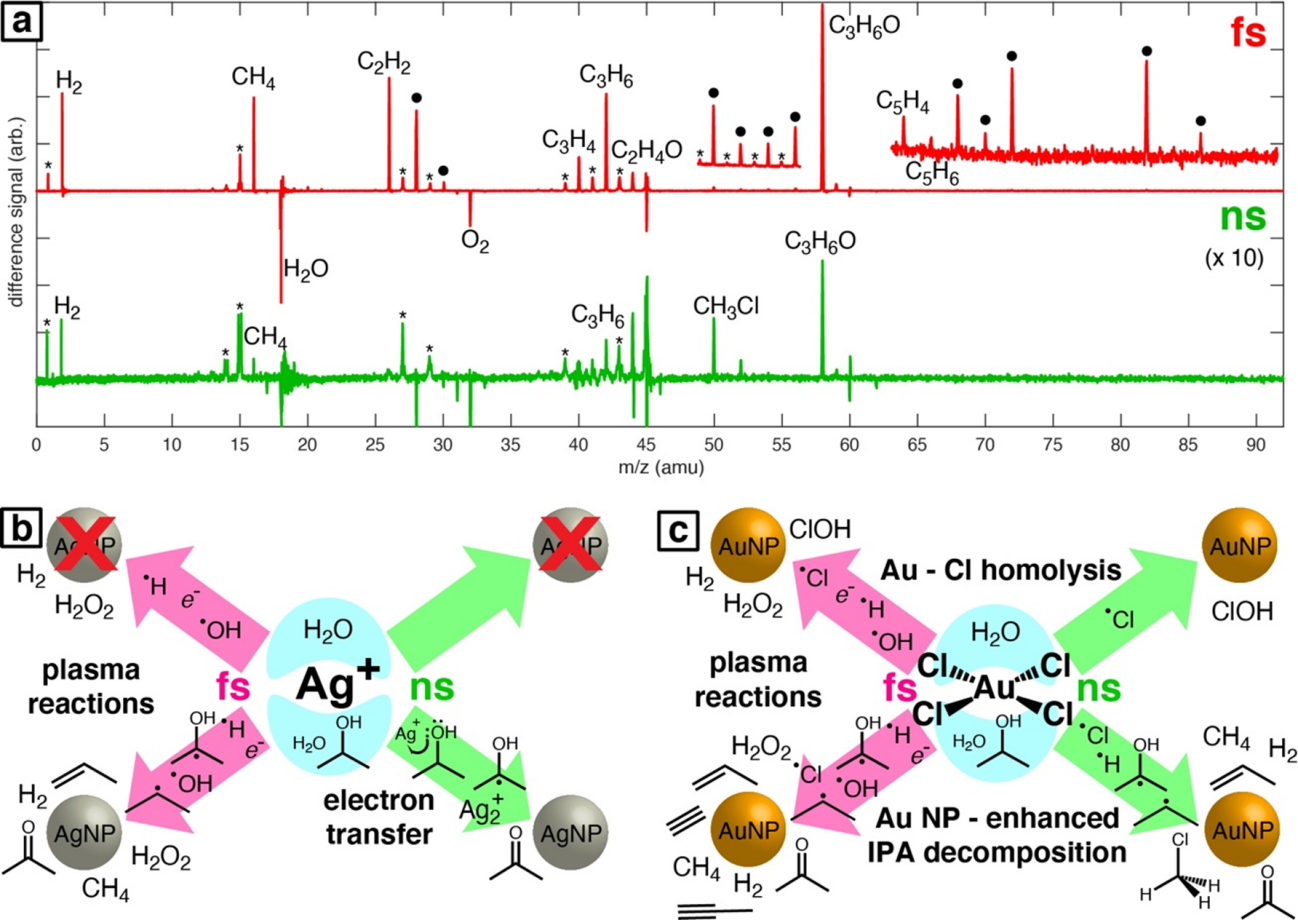
a) Laser mass spectra obtained after femtosecond (red) and nanosecond (green) LRL of AuCl_4_^−^ in a water–isopropyl alcohol mixture with major products indicated. The • denotes that multiple species can contribute to this peak and the * indicates a peak due to fragmentation. The ns-LRL spectrum is magnified by a factor of ten. Insets magnify the regions *m*/*z* 49–57 and 63–91 in the fs-LRL spectrum to show low-yield products. Schematic of different reaction pathways for the LRL of (b) Ag^+^ and (c) AuCl_4_^−^ and detected reaction products and reaction intermediates indicated for each reaction condition: femtosecond (pink) or nanosecond (green) laser excitation; water (top) or water–isopropyl alcohol (bottom). Figure 6 was reproduced from [121] (“Understanding photochemical pathways of laser-induced metal ion reduction through byproduct analysis”, © 2023 L. M. F. Batista et al., published by The Royal Society of Chemistry, distributed under the terms of the Creative Commons Attribution-NonCommercial 3.0 Unported License, https://creativecommons.org/licenses/by-nc/3.0/). This content is not subject to CC BY 4.0.

However, hydrogen is not the only gas observed during nanoparticle formation processes. Kalus et al. reported the formation of CO and CO_2_, along with CH_4_ and C_2_ hydrocarbons [106]. Further, almost no O_2_ is formed during the ablation of gold in ethylene glycol, resulting in a conversion of chemically bound oxygen to possible molecular non-volatile products and/or CO and CO_2_. Frias Batista et al. recently investigated the chemical reactions occurring during the LRL of AuCl_4_ and AgClO_4_ in water–isopropyl alcohol solution using femtosecond and nanosecond lasers (Figure 6).

Isopropyl alcohol was found to decompose to numerous products, including methane, acetylene, propene, and C_5_ alkyne hydrocarbons. However, the product yields found during laser mass spectrometry measurements were higher for fs-LRL than for ns-LRL, indicating that femtosecond irradiation enhances chemical reactions of the solvent, possibly caused by laser-induced shockwaves or optical breakdown of the liquid. The decomposition of isopropyl alcohol was enhanced by the Au nanoparticles resulting from LRL, producing the previously mentioned C_1_–C_5_ products. In addition to this, the authors found a difference regarding the nucleation of Ag nanoparticles depending on the used solvent. For the formation of Ag nanoparticles during LRL in isopropyl alcohol, they proposed a mechanism of electron transfer from the solvent, which produced acetone as a by-product. Since this transfer is not possible in water and only plasma reactions are available, Ag could not nucleate during LRL in water because of the oxidizing activity of hydroxyl radicals [121]. Overall, LSPC in organic solvents leads to higher gas volumes and a more complex gas mixture, consisting of hydrogen and highly volatile hydrocarbons, than in water. The gas and hydrogen formation rates were also shown to be connected to the pyrolysis processes and chemical properties of the used solvent. Furthermore, LSPC in organic solvents enables different reaction pathways compared to water and, hence, allows the formation of different nanoparticle products.

#### Hydrocarbon formation

While the production of gases might initially appear unrelated to the widespread observations of carbon shells surrounding nanoparticles produced by LSPC in organic solvents, another category of carbon-based molecules is formed that might link the two species, namely pyrolysis products. Pyrolysis products were previously found during photolysis of organic solvents [52,117] as well as laser ablation [122–123], laser fragmentation [124], laser melting [125], and laser reduction [120–121]. While the formation of short hydrocarbons during laser-based synthesis of nanoparticles in organic solvents is known, the amount of literature in this regard is scarce. An overview of formed hydrocarbons is given in Table 1.

**Table 1:** Molecular hydrocarbons formed through laser ablation, laser fragmentation, laser reduction, and laser melting in organic solvents or irradiation of pure solvents.

Process and used material	Used solvent	Formed by-products	Ref.

Au_LAL_	glycols	CH_4_, C_2_H_6_, C_2_H_4_, C_2_H_2_, CO, CO_2_, H_2_	[106]
Zn_LAL_	tetrahydrofuran	olefinic and carbonyl species	[123]
Al_LAL_	acetone	enolates and carboxylates	[122]
Nd_2_Fe_14_B_LFL_	cyclohexane	C_2_–C_7_ hydrocarbon fragments	[124]
Fe_LML_	ethanol	C_2_H_4_O, C_4_H_10_	[125]
Fe_LML_	ethyl acetate	C_2_H_4_O, C_4_H_10_	[125]
Cu_LML_	ethanol	CO_2_, CO, CH_4_, C_2_H_6_, C_2_H_4_	[126]
Cu(OAc)_2,LRL_	acetonitrile	CH_4_, HCN, H_2_	[120]
Cu(OAc)_2,LRL_	propionitrile	CH_4_, HCN, H_2_	[120]
Cu(OAc)_2,LRL_	benzonitrile	CH_4_, H_2_	[120]
KAuCl_4_, AgClO_4,LRL_	2-propanol	CH_4_, C_2_H_2_, C_3_H_4_, C_3_H_6_, C_2_H_4_O, C_5_H_4_, C_5_H_6_	[121]
Solvent_LRL_	C_5_–C_11_ alkanes	dimers, trimers, C_4_ to C_9_ species	[52]
Solvent_LRL_	octane	polyynes (up to C_14_), C_2_H_2_, C_2_H_4_, C_3_H_4_, H_2_	[119]
Solvent_LRL_	benzene	biphenyl, terphenyl, styrene, and many more	[115]
Solvent_LRL_	benzene	H_2_, CH_4_, C_2_H_2_, C_2_H_4_, toluene, (methyl-)biphenyl, phenanthrene, (methyl-)anthracene	[116]
Solvent_LRL_	cyclohexane	CH_4_, C_2_H_2_, C_2_H_4_, C_2_H_6_	[116]
Solvent_LRL_	toluene	biphenyl, anthracene, pyrenes	[116]
Solvent_LRL_	toluene	C_2_H_2_, C_2_H_4_, C_3_H_4_, C_4_H_2,_ C_8_H_2_, acetophenone, benzyl alcohol, dimers, phenylacetylene, naphthalenes, fluorene	[117]
Solvent_LRL_	ethanol	CH_4_, C_2_H_2_, C_2_H_4_, C_3_H_4_, C_3_H_6_, C_4_H_4_, dimers	[117]
Solvent_LRL_	acetone	CH_4_, C_2_H_2_, C_2_H_4_, C_3_H_4_, C_3_H_6_, acetylacetone, dimers	[117]
Solvent_LRL_	*n*-hexane	CH_4_, C_2_H_2_, C_2_H_4_, C_3_H_4_, C_3_H_6_, C_4_H_2_, C_4_H_4_, C_4_H_6_, C_6_ alcohols and ketones, C_9_–C_11_ alkanes, dimers, styrene, phenylacetylene, naphthalenes	[117]

**Figure 7:**
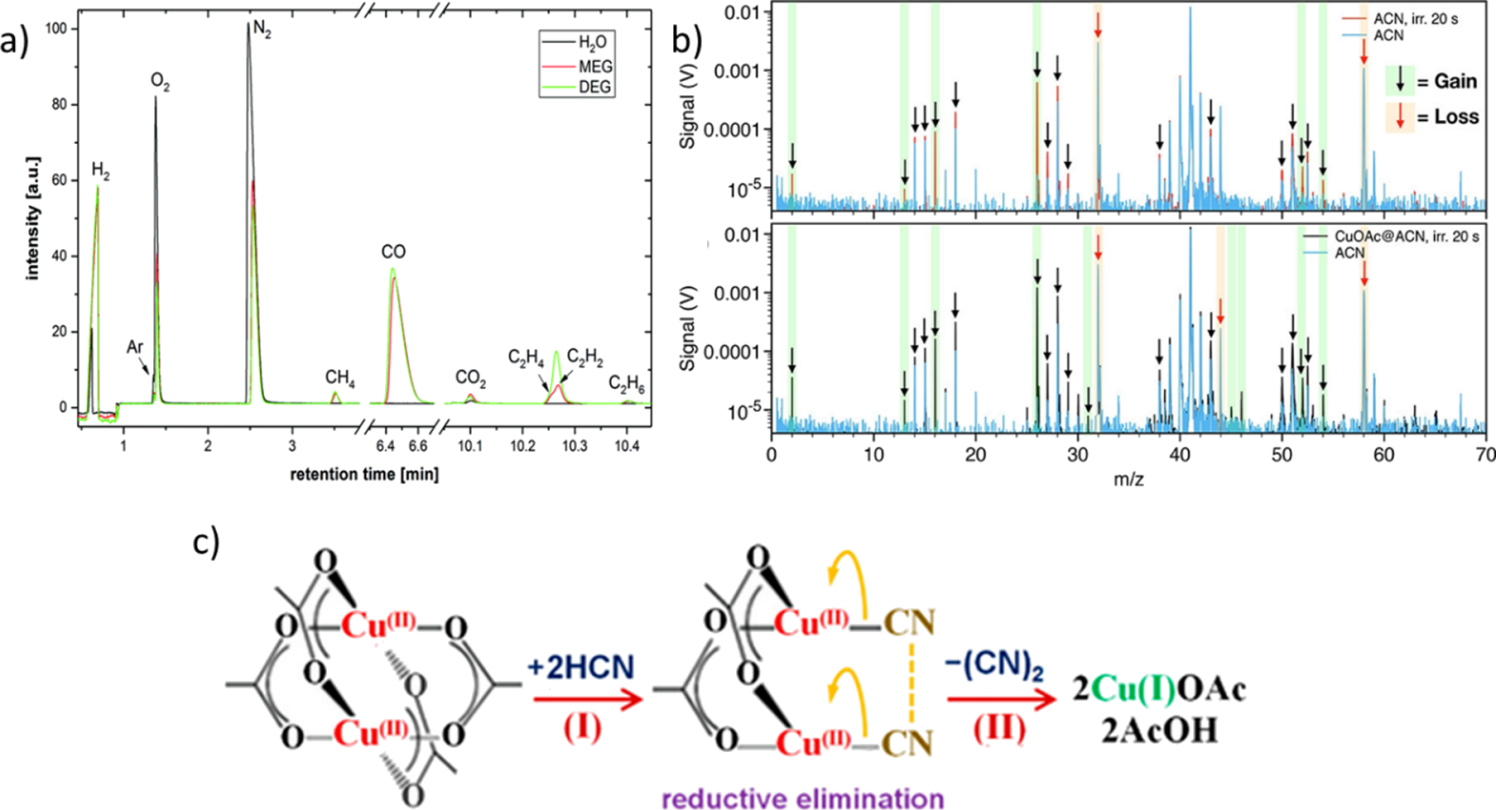
(a) Gas chromatograms (GC-TCD) showing the production of hydrogen and oxygen during LAL in water (black line) and the formation of hydrogen, carbon monoxide, carbon dioxide, and other hydrocarbons during LAL in glycols (green and red lines). Figure 7a was republished with permission of PCCP Owner Societies, from [106] (“How persistent microbubbles shield nanoparticle productivity in laser synthesis of colloids – quantification of their volume, dwell dynamics, and gas composition” by M.-R. Kalus et al., *Phys. Chem. Chem. Phys.*, vol 19, issue 10, © 2017); permission conveyed through Copyright Clearance Center, Inc. This content is not subject to CC BY 4.0. (b) Mass spectra of headspace gas of unirradiated acetonitrile (blue) and irradiated acetonitrile without copper acetate (red, top) and with copper acetate (red, bottom). (c) Schematic depiction of the reaction between copper(II) acetate dimer with HCN leading to the formation of copper(I) acetate through reductive elimination of CN-CN. Figure 7b, c was reprinted with permission from [120], Copyright 2019 American Chemical Society. This content is not subject to CC BY 4.0.

Kalus et al. also reported the formation of CH_4_, C_2_H_2_, C_2_H_4_, and C_2_H_6_ during ns-LAL [106] (Figure 7a). Similar results (but for a totally different LSPC process) were published by Tangeysh et al., who performed fs-LRL of copper salts in acetonitrile, propionitrile, and benzonitrile. Besides the formation of methane, mass spectrometry measurements of the obtained solvents showed the formation of HCN and propionitrile. HCN formation did not occur during LRL in benzonitrile, which was ascribed to the lack of α-hydrogen in the solvent molecules. The authors further proposed the formation of CuCN-polyacetonitrile chains during the reduction step [120]. LML of iron oxide in ethyl acetate and ethanol was reported to yield ethyl aldehyde and butane. The aldehyde was proposed to form via dehydrogenation of ethanol, while butane forms via dimerization of formed C_2_H_5_ radicals [125]. Van’t Zand et al. investigated the pyrolysis of tetrahydrofurane (THF) at a total energy input of 2250 J comparing fs-, ps-, and ns-pulsed lasers using FTIR spectroscopy. They found a significant decrease in the dominant C–O–C bond at 1070 cm^−1^ after ablation, which indicates cleavage of the C–O–C bonds in THF. C–H and C–C bonds were detected before and after ablation, while at wavenumbers between 1650 cm^−1^ and 1725 cm^−1^ signals could be detected after the ablation process. Bonds at those wavenumbers represent olefinic species or carbonyl groups possibly formed during the C–O–C bond cleavage. Apparently, fewer olefinic and more carbonyl products are formed when using nanosecond lasers compared to picosecond and femtosesond lasers [123]. In general, the irradiation of organic solvents (during LSPC) leads to the formation of various by-products, also through pyrolysis reactions. While formed substances with short carbon chains are gaseous, the formation of liquid compounds, which include saturated, unsaturated, and aromatic hydrocarbons, is also possible. Furthermore, the pulse duration affects the quantity of the generated by-products; ultrashort pulses (picoseconds and femtoseconds) yield larger amounts than nanosecond pulses.

**Figure 8:**
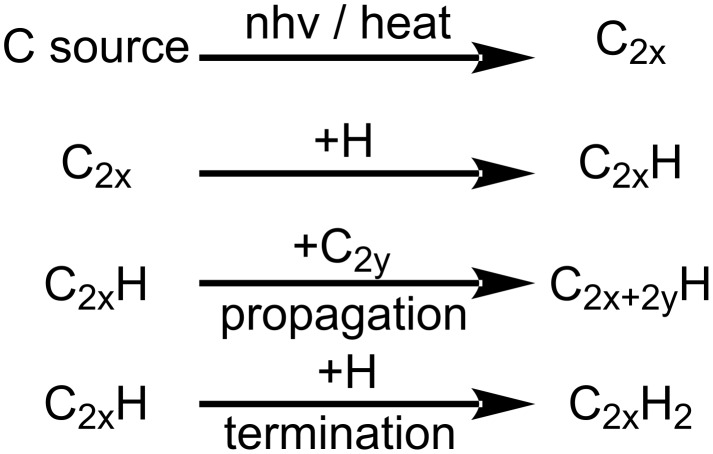
Scheme of the formation of hydrogen-capped polyynes during ns-LAL. The carbon source can be either a carbon-based target or an organic solvent. Figure 8 was redrawn from [132].

Not only was the formation of sp^2^- or sp^3^-hybridized short-chain hydrocarbons observed but also the formation of sp-hybridized polyynes, which are linear hydrocarbons consisting of alternating single and triple bonds. They are often synthesized by laser ablation of carbon-based materials in water [127–131] or organic solvents [130,132–133] with carbon chain lengths of up to 26 [133]. However, they are also generated when irradiating pure organic solvents [117,134–137] or during the ablation of metals [131,138–139]. Pan et al. synthesized polyynes with a carbon chain length of ten by ablation of gold in ethanol. They proposed that both the gold target and ethanol have a fundamental impact on the carbyne formation. While gold catalyzes the dehydrogenation of ethanol to form carbon–carbon triple bonds, the structure of ethanol utilizes the C_2_ carbon chain as a building block of the carbyne chain and the hydroxyl group forms the initial Au–H adduct needed for the catalytic reaction. Other alcohols, such as methanol or propanol, did not lead to carbyne formation during the experiments [139]. Condorelli et al. were able to synthesize carbon-encapsulated Pt nanoparticles by RLAL of graphite in a colloidal Pt solution [138]. The chemical processes leading to the formation of polyynes are still not fully understood, but a few models have been proposed over the years. Tsuji et al. proposed a model for ns-LAL. They suggested a stepwise growth of hydrogen-capped polyynes by the addition of carbon radical fragments, depicted in Figure 8. This radical propagation process competes with the hydrogenation reaction, which terminates the elongation reaction [132]. The carbon source for this polymerization reaction is either the ablated material or decomposed solvent molecules [140]. Besides the termination reaction with hydrogen, different end-capping groups such as CN or CH_3_ can be added [118,132,140–143].

While this mechanism for polyyne formation is widely accepted for ns-LAL, the synthesis of polyynes by fs-LAL is proposed to undergo different reactions. In contrast to ns-LAL, where elemental carbon is used as a source to form the initial polyyne fragments, fs-LAL reaches power densities that enable direct ionization and dissociation of the solvent, which may form ionized, short polyynes or cumulenes without intermediate steps. Short C_4_ polyynes have been observed by femtosecond laser mass spectrometry of irradiated organic solvents [117,119]. Long-chained polyynes, however, cannot be formed this way and, hence, require follow-up reactions of the short cumulenes [119,135–137]. The difference between the proposed mechanisms for ns-LAL and fs-LAL is that Tsuji et al. [132] proposed a radical propagation mechanism, while the fs-LAL mechanism proposed by Zaidi et al. uses ionic propagation [119]. In general, the necessary species for both formation mechanisms are either elemental carbon or solvent fragments, depending on the used pulse width. In this regard, laser fluence is crucial for the synthesis of polyynes. On the one hand, if the fluence is below a certain threshold for solvent decomposition, the necessary fragments and, hence, polyynes cannot be formed. On the other hand, if the fluence is too high or the irradiation time is too long, processes leading to diamond-like structures and graphitization during the LAL process are favored, resulting in a quick formation of graphite and, thus, no available fragments for the polyyne formation [101,144–146]. Considering these factors, the polyyne yield should be highly influenced by the applied fluence and undergo a maximum at a given fluence, which decreases afterward because of faster graphitization steps or in situ destruction of the generated polyyne structure. Marabotti et al. performed ablation processes at different fluences, quantified the concentration of different C_8_ polyynes and found the previously mentioned correlation: First, an increase in fluence results in a steep rise in polyyne concentration. However, at a given point, a maximum is passed, and the polyyne concentration decreases again and remains at a rather constant level [64]. As short-chained C_8_ polyynes were investigated, the curve starts increasing at a given fluence. However, since the synthesis of the longest-chain polyyne (HC_30_H) ever produced used a fluence of 0.57 J·cm^−2^ [147], one can assume that higher fluences favor short-chained polyynes since long-chained polyynes tend to decompose. In turn, lower fluences yield long-chained polyynes as they are not destroyed. To recapitulate, LSPC can be accompanied by the formation of polyynes, which only consist of sp-hybridized carbon atoms with chain lengths of up to 26. The carbon source stems from either the liquid or the target material. The mechanism for ns-LAL polyyne formation can be retraced to the polymerization of C_2_ building blocks and can be partially controlled by varying the fluence. In contrast, fs-LAL enables ionization of the solvent to directly generate short polyynes, which elongate by follow-up reactions. A more elaborate description of the mechanism processes related to polyynes can be found in the recent review article by Marabotti and coworkers [64].

#### Solid carbon formation and nanoparticle surface modification

**Figure 9:**
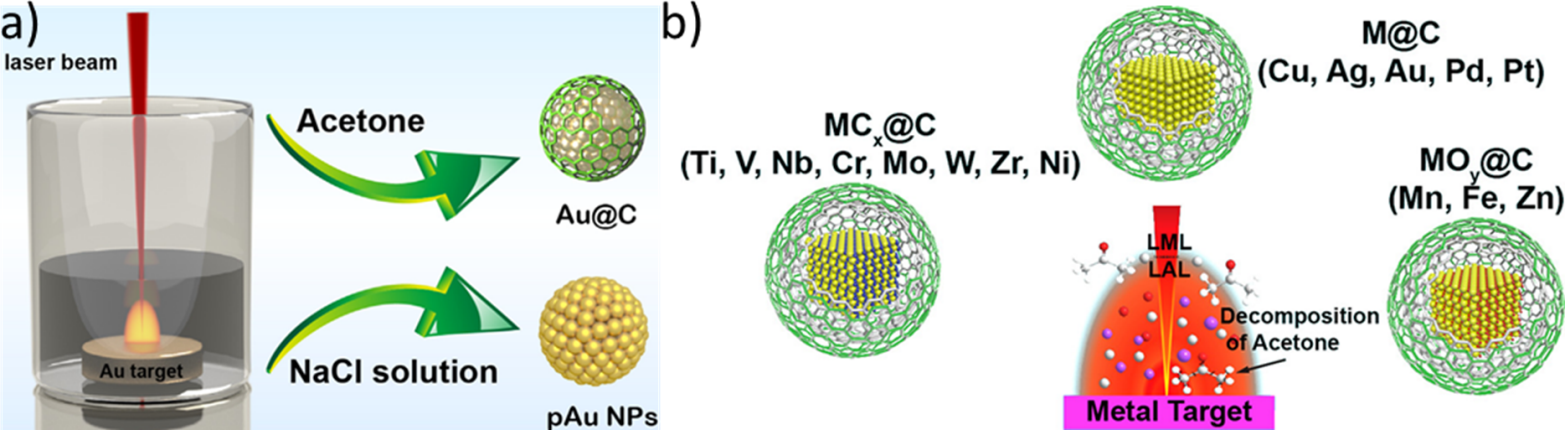
Carbon shell formation typology during LSPC in organic solvents. (a) Illustration of the laser ablation of gold in acetone and NaCl solution and the nanoparticles’ properties. Figure 9a was republished with permission of Elsevier, from [155] (“Laser-synthesized graphite carbon encased gold nanoparticles with specific reaction channels for efficient oxygen reduction” by C. Zhang et al., *Journal of Colloid and Interface Science*, vol 563, © 2019); permission conveyed through Copyright Clearance Center, Inc. This content is not subject to CC BY 4.0. (b) Formed core–shell structures of nanoparticles generated through the LAL of metals in acetone. Figure 9b was reprinted with permission from [101], Copyright 2019 American Chemical Society. This content is not subject to CC BY 4.0.

While the oxidation of nanoparticles formed by LSPC can be attributed to redox reactions that result in water splitting [50–51], the mechanism for the carbon shell formation during LSPC in organic solvents is still unclear. In this context, different mechanisms were postulated; they mostly begin with the thermal decomposition of the organic solvents into either molecular carbon or hydrocarbon fragments. Currently, it is proposed that either atomic or molecular fragments from the solvent mix into the ablated matter and segregate to the surface followed by carbonization steps [148] or that molecular (hydro)carbon species adsorb and condensate on the surface and carbonize to form the carbon shells [149]. The latter results in a dependency of carbon shell thickness on particle size, which was modeled by Reichenberger and coworkers [54]. In detail, during LSPC of inorganic nanoparticles in organic solvents, carbon is formed in addition to permanent gases or molecular carbon-based species. While the surface of colloids synthesized in water is only modified by the surfactants (or inorganic anions) used, the carbon stemming from the organic solvent molecules acts as a surface modification of the nanoparticles and is often obtained as carbon shells. The carbon shell can be amorphous carbon [100–101], graphitic carbon [104,149–152], or carbide [99,101,153–154], depending on the metal’s properties (i.e., carbon affinity). Zhang et al. researched the metal’s influence on the formed carbon species by ablation of 16 different transition metals in acetone and categorized the obtained core–shell structures into three classes depending on the ablated metal (Figure 9b) [101]. The first class consists of inert metals (Cu, Ag, Au, Pd, and Pt), which form an elemental metal core with a graphitic carbon shell. Active metals with stable carbides (Ti, V, Nb, Cr, Mo, W, Zr, and Ni) make up the second class and form mixed metal/metal carbide/metal oxide cores and graphitic carbon shells. The third group consists of metals with metastable carbides (Mn, Fe, and Zn). As their carbides are either unstable or the metals have a higher affinity to oxygen than to carbon, the generated nanoparticles consist of metal/metal oxide cores and carbon shells. These carbides might form in situ but decompose during the process to yield an oxide core.

Iron oxide is not the only phase formed in organic liquids during laser ablation of iron (Table 2). Amendola et al. ablated iron in six different solvents and found different core compositions. Ablation in toluene gave rise to an amorphous iron core, while tetrahydrofuran and dimethyl sulfoxide formed magnetite/maghemite or metallic iron. Oxides were detected for acetonitrile and dimethylformamide, and ablation in ethanol resulted in iron carbides [100]. Iron carbide formation was also found for ablation in alkanes (pentane, hexane, and decane) in an inert atmosphere by Matsue and coworkers [102]. Hence, the formed phase of iron nanoparticles is determined by the chemical properties of the organic solvent and the used atmosphere, which is closely related to the formed decomposition species, which can be of reductive nature (e.g., hydrogen).

**Table 2:** Compositions of iron-based nanoparticles formed through laser ablation of iron in organic solvents.

Solvent	Formed core composition	Wavelength	Pulse duration	Ref.

tetrahydrofuran	metallic Fe	1064 nm	9 ns	[100]
dimethyl sulfoxide	metallic Fe	1064 nm	9 ns	[100]
toluene	amorphous Fe	1064 nm	9 ns	[100]
acetone	metallic Fe/oxide	1064 nm	7 ns	[101]
acetonitrile	oxide	1064 nm	9 ns	[100]
dimethylformamide	oxide	1064 nm	9 ns	[100]
ethanol	carbide, oxide	1064 nm	9 ns	[100]
pentane	carbide	532 nm	4–7 ns	[102]
hexane	carbide	532 nm	4–7 ns	[102]
decane	carbide	532 nm	4–7 ns	[102]

Depending on the nature of the organic solvents, a modification of the nanoparticles or the carbon shell is possible, including doping with heteroatoms from the solvent molecules. Jung et al. produced different phases of Ni nanoparticles in hexane and acetone [156], and the ablation of Pb, Zn, and Cu was performed by Niu et al. in thiols to gain the respective sulfides [157]. In contrast to the change of the nanoparticle core, Choi et al. elegantly obtained N-doped carbon shells or nitrides when they ablated in acetonitrile [104–105]. A change of solvent to propionitrile or butyronitrile, however, led to the formation of regular (heteroatom-free) graphitic carbon shells. Begildayeva et al. attributed this behavior to the high availability of the CN^−^ moiety in acetonitrile after decomposition, while the decomposition of nitriles with longer chains leads to the release of alkyl carbons [105].

**Figure 10:**
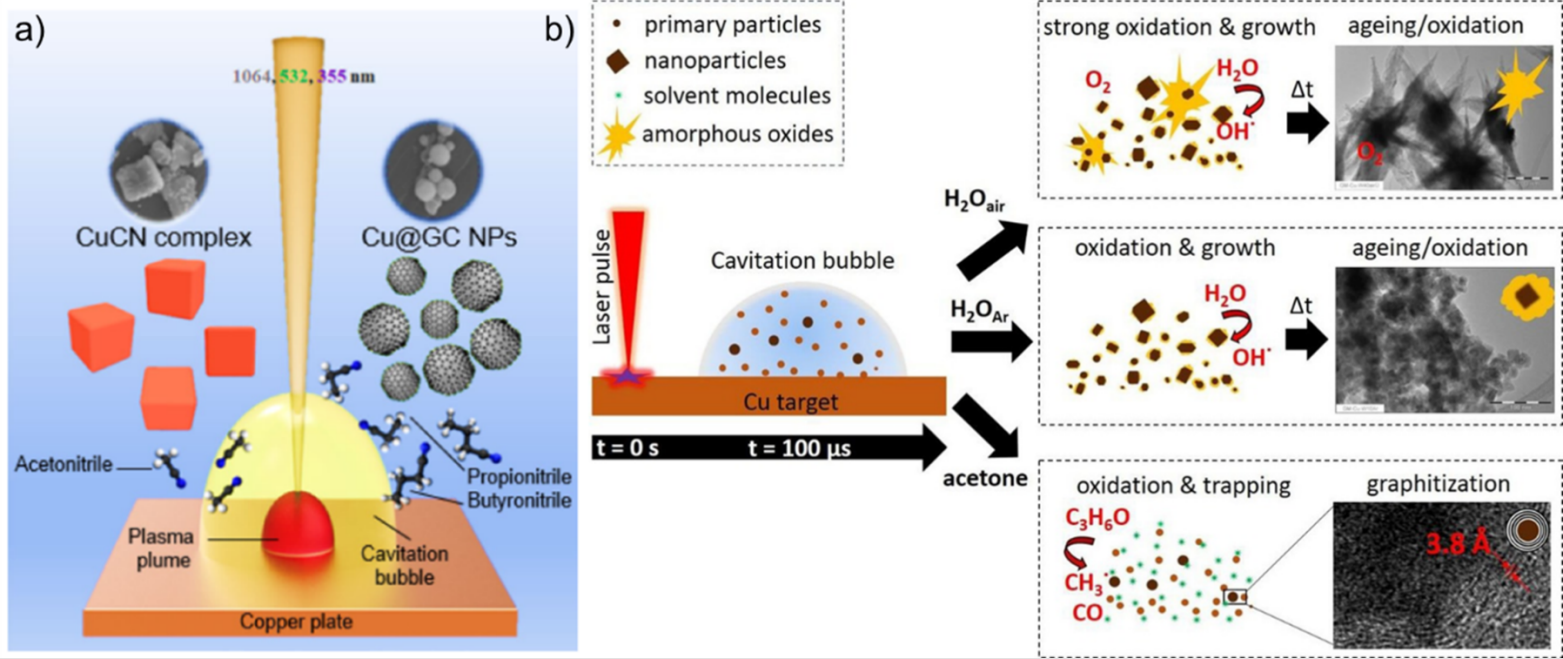
Behavior of copper during LSPC in organic solvent. (a) LAL of a Cu target using wavelengths of 1064, 532, or 355 nm in various nitrile solvents (acetonitrile, propionitrile, or butyronitrile leading to the formation of cubic CuCN particles for acetonitrile and spherical Cu@GC particles for propionitrile and butyronitrile.Figure 10a was reproduced from [105] (© 2021 T. Begildayeva et al., published by Springer Nature, distributed under the terms of the Creative Commons Attribution 4.0 International License, https://creativecommons.org/licenses/by/4.0). (b) Synthesis of Cu/CuPt nanoparticles by PLAL in H_2_O in air, H_2_O in Ar atmosphere, and acetone. Figure 10b was republished with permission of Wiley, from [86] (“Role of Dissolved and Molecular Oxygen on Cu and PtCu Alloy Particle Structure during Laser Ablation Synthesis in Liquids” by G. Marzun et al., *ChemPhysChem*, vol. 18, issue 19, © 2017 Wiley-VCH Verlag & GmbH & Co. KGaA, Weinheim); permission conveyed through Copyright Clearance Center, Inc. This content is not subject to CC BY 4.0.

In addition to this, nanoparticle oxidation can be reduced in organic solvents compared to water. Marzun et al. found strong oxidation of Cu particles in water, while the degree of oxidation for Cu particles synthesized in acetone was lower (Figure 10) [86]. Khairani et al. found comparable results for the ablation of FeNi alloys in water and acetone, reducing the overall oxygen content on the nanoparticles’ shell surface from 28 atom % to 18 atom % [98]. A change of atmosphere from air to Ar as inert gas or hydrogen as reductive gas was reported to reduce the oxidation of the nanoparticles even further [97,120]. In summary, the properties of atmosphere, solvents, and metals strongly influence the obtained nanoparticles. If the metals have an affinity to bind carbon, the formation of carbides is possible, but not ensured. In case of iron, non-functionalized hydrocarbons such as *n*-hexane seem to favor carbide formation, while other solvents lead to metallic or amorphous iron or iron oxide. Furthermore, the solvent influences the properties of the carbon shell by enabling, for example, nitrogen doping.

**Figure 11:**
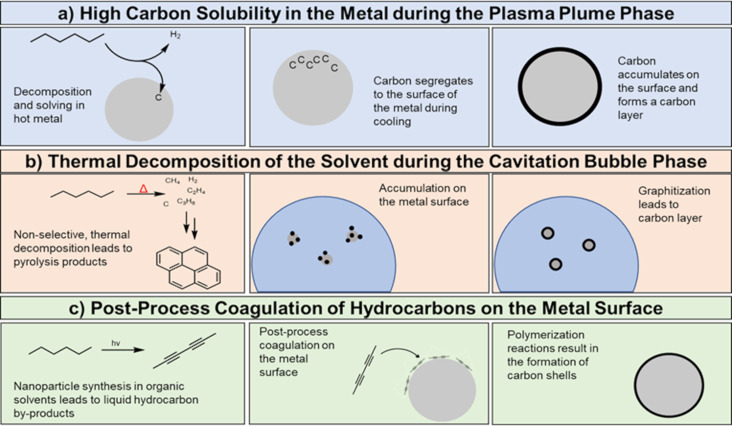
Current hypotheses on the carbon shell formation during LSPC in organic solvents. (a) Solvent decomposition and subsequent solvation of carbon in the hot metal postulated by Choi and coworkers. Figure 11a was redrawn from [104,149]. (b) Thermal pyrolysis of the solvent during the cavitation bubble phase postulated by Zhang and coworkers. Figure 11b was redrawn from [101]. (c) Post-process condensation of hydrocarbons or polyynes on the metal surface postulated by Compagnini and coworkers. Figure 11c was redrawn from [158].

Currently, there are three different hypotheses for the formation mechanism of the carbon shells schematically shown in Figure 11: (i) Choi et al. proposed a decomposition of the solvent during the plasma phase and a subsequent (partial) solution of carbon in the ablated metal depending on the solubility of carbon in the molten metals (Figure 11a). This would result in higher carbon shell thicknesses for larger nanoparticles due to the decrease in surface-to-volume ratios of the nanoparticles [104,149]. Reichenberger et al. proposed a model formula (Equation 1) for the ablation in acetone to calculate the volume-specific nanoparticle surface area *S*_NP_ and the nanoparticle diameter *d*_NP_ and correlated it successfully with experimental results (Figure 11a) [54].


[1] $$S_{\text{NP}}=\frac{\text{A}[{\text{m}}^{2}]}{\text{V}[{\text{cm}}^{3}]}=6000*{\left(d_{\text{NP}}\left[\text{nm}\right]\right)}^{-1}$$

Choi and Jung performed further investigations regarding the carbon solubility in the metal by ablating Au in methanol, *n*-hexane, acetonitrile, and water. They performed acid resistance tests to confirm the presence of carbon shells and found none for Au nanoparticles synthesized in methanol and water. The nanoparticles generated in *n*-hexane and acetonitrile, however, were found to possess graphitic carbon shells on the surface up to a particle diameter of 50 nm as the size distribution after the acid treatment found only particles with a size below 50 nm that resisted acid dissolution. They proposed the carbon solubility in the Au nanoparticles to be the reason for the coverage of particles with a size below 50 nm and calculated the carbon solubility as a function of particle radius according to Equation 2. This yielded the carbon solubility *S* as a function of the particle radius *r* via the solubility in the bulk state *S*_0_, the surface tension σ, the volume of a metal molecule *V*, the Boltzmann’s constant *k* and the melting temperature of the bulk state *T*. When the Au particle size exceeds a threshold, the maximum amount of solute carbon in the particle exceeds the actual amount of dissolved carbon; therefore, carbon does not segregate to the surface, resulting in a bare nanoparticle superficies that is not resistant to acids [149].


[2] $$S=S_{0}\exp\left(\frac{2\sigma V}{kTr}\right)$$

**Figure 12:**
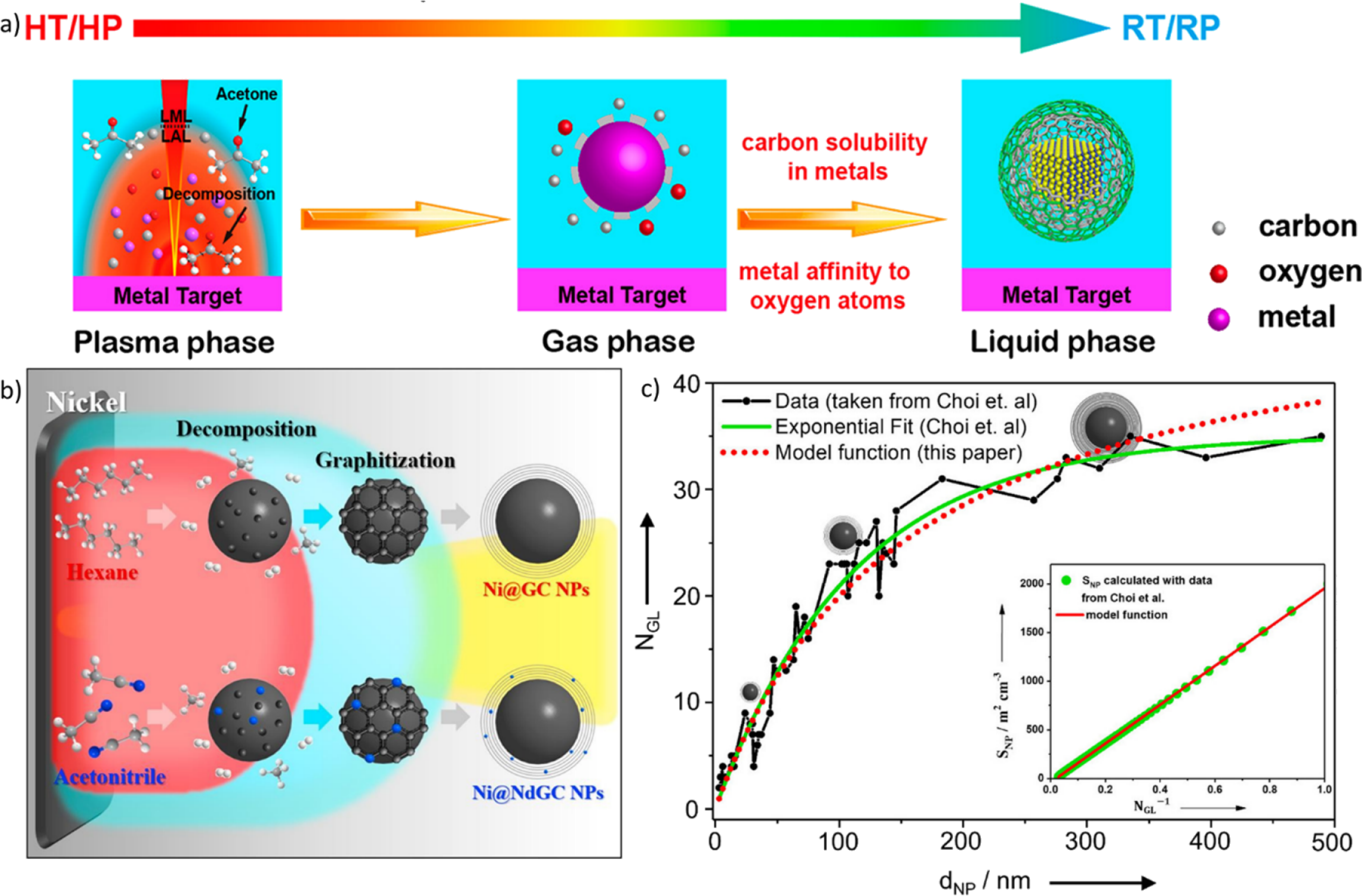
(a) Quenching process during PLAL of metals in acetone and the proposed formation mechanism of different carbon-related nanoparticle products (M@C, MC*_x_*@C, and MO*_y_*@C) by carbon solution and segregation in molten metals. HT: high temperature, HP: high pressure, RT: room temperature, RP: room pressure. Figure 12a was adapted with permission from [101], Copyright 2019 American Chemical Society. This content is not subject to CC BY 4.0.(b) Schematic formation mechanism of Ni@GC and Ni@NdGC by solvent decomposition during the cavitation bubble phase. GC: graphitic carbon, NdGC: N-doped graphitic carbon. Figure 12b was republished with permission of Elsevier, from [104] (“One-pot synthesis of graphitic and nitrogen-doped graphitic layers on nickel nanoparticles produced by pulsed laser ablation in liquid: Solvent as the carbon and nitrogen source” by H. J. Jung et al., *Applied Surface Science*, vol 457, © 2018; permission conveyed through Copyright Clearance Center, Inc. This content is not subject to CC BY 4.0.(c) Correlation of the number of formed graphene layers (*N*_GL_) around NiNP as a function of the NiNP diameter (*d*_NP_) generated by LAL in acetonitrile with the raw data and exponential fit by Reichenberger and coworkers. Figure 12c was republished with permission of Wiley, from [54] (“Perspective of Surfactant-Free Colloidal Nanoparticles in Heterogeneous Catalysis” by S. Reichenberger et al., *ChemCatChem*, vol. 11, issue 18, © 2019 Wiley-VCH Verlag GmbH & Co. KGaA, Weinheim) and regarding the green and black curve also of Elsevier, from [104] (“One-pot synthesis of graphitic and nitrogen-doped graphitic layers on nickel nanoparticles produced by pulsed laser ablation in liquid: Solvent as the carbon and nitrogen source” by H. J. Jung et al., *Applied Surface Science*, vol 457, © 2018); permission conveyed through Copyright Clearance Center, Inc. This content is not subject to CC BY 4.0.

However, this correlation has been validated only for the researched Au nanoparticles and used laser conditions, yet. To propose a general equation to calculate the amount of solved carbon and, hence, formed graphitic carbon shells, further investigations need to determine the amount of carbon formed, the carbon solubility in the solid and the liquid metal, the catalytic effect to reduce carbon, and the tendency to form stable carbides depending on the ablated metal, the used solvent, and the applied laser parameters. (ii) In contrast to the carbon supersaturation–surface excretion mechanism, Zhang et al. attributed the carbon shell formation to the plasma/cavitation bubble phase in which the carbon-based solvent is decomposed, followed by an accumulation of the decomposition products on the metal surface and a final carbonization step to form carbon shells on the nanoparticle surface [101,148]. The latter were reported to undergo graphitization to onion-like shells during post-treatment irradiation (Figure 11b, Figure 12) [101]. (iii) Compagnini et al. [158] as well as Zhang et al. [159] reported slowly growing carbon shells on nanoparticle surfaces. Hence, a third mechanism may be proposed (Figure 11c). In detail, Compagnini et al. found growing carbon structures on top of surfactant-free gold, silver, and copper nanoparticles after adding polyynes, while Zhang et al. performed ablation of silver in acetone with long-term storage, which resulted in shape alteration, size separation, and changes in the carbon shells. As such, molecular hydrocarbons with unsaturated bonds might accumulate over longer periods on the nanoparticle surface and form carbon via polymerization steps afterward. In this regard, the previously mentioned polyynes must be highlighted as long polyyne chains are inherently unstable due to their ability to cross-link with each other to produce graphite structures. The carbon shell is a surface modification and, as such, affects the nanoparticles’ catalyst properties either positively [37,155] or negatively [149]. A low number of graphene layers on the surface of the nanoparticles was found to enhance the catalytic activity during alkaline hydrogen evolution reactions [160]. This was partly attributed to an optimization of water adsorption and stabilization of *H and *OH, which leads to an acceleration of reaction kinetics [161]. As the structure and thickness of the carbon shell directly affect the properties of nanoparticles, further research on the formation mechanism is crucial for the utilization of laser-synthesized nanoparticles.

Although solid carbon has been observed most widely in the form of carbon shells around inorganic nanoparticles formed by LAL, as discussed above, other LSPC processes also produce solid carbon. Irradiation of organic solvents in the absence of inorganic targets has been observed to form solid carbon nanoparticles, as reviewed recently by Frias Batista and coworkers [43]. For instance, fluorescent carbon dots, which are amorphous carbon-based particles with a diameter below 10 nm, are obtained upon femtosecond and picosecond irradiation of solvents including toluene, hexane, acetone, and acetonitrile [117,162–163]. Nanodiamonds have been obtained through femtosecond irradiation of ethanol [164–165]. Fluorescent carbon dots were also observed as a by-product when synthesizing 1-octene-capped silicon nanoparticles by picosecond LAL [166], indicating that solid carbon products can form independently from solvent decomposition during LAL. Femtosecond LRL of copper and silver acetylacetonate in alcohol solvent was found to produce Cu and Cu–Ag alloy nanoparticles with carbon shells composed of disordered graphite oxide [37,167]. However, the carbon shells were found to grow around the Cu nanoparticles after termination of the laser irradiation, suggesting that the copper nanoparticle surfaces catalyzed the condensation of reactive solvent decomposition products into the observed carbon shells. In summary, the mechanism of how carbon shells form is still under intensive discussion, but all three of the mechanisms mentioned above have at least been partially demonstrated. As such, the carbon shells form either via (i) solvation of carbon in the hot metal droplet/particle with subsequent segregation to the nanoparticle surface during cooling, (ii) decomposition of the solvent molecules on the (hot) particle surface during the process, or (iii) post-process adsorption and accumulation of formed carbon by-products on the particle surface.

### Theoretical and conceptual approaches to laser-induced solvent decomposition

Reductive species are expected to be formed during the laser-based synthesis of nanoparticles. However, the (thermal or photochemical) decomposition of solvents, including the post-condensation or redox reactions of the decomposition products, is currently a black box. Although the formation of amorphous, graphitic, and carbide carbon, as well as hydrogen has been demonstrated, the intermediates that lead to carbon and hydrogen formation have rarely been addressed. The decomposition of the solvents must be initiated due to the unique process parameters reaching high temperatures of several thousand kelvins and pressures up to the gigapascal range that prevail during nanoparticle synthesis [58,168–169]. Furthermore, the Nernst potential is temperature-dependent, increasing the reactivity of metal precursor and formed nanoparticles during the process. In this context, an increase or decrease in temperature (along with the heating/cooling rate) during nanoparticle formation should lead to a quantitative and qualitative change in the decomposition products since the temperatures are equivalent to the energy available for the decomposition reactions. The main process parameter influencing the temperature during nanoparticle synthesis is the laser fluence, which was shown to also induce optical breakdown of the liquid during LSPC [49,116,170]. Consequently, an increase in laser fluence should lead to increased formation and altered distribution of the by-products, either by breakdown of the solvent or an excess in energy during particle formation. This was discussed earlier for the polyyne formation, where higher fluences would lead to a decrease in polyyne concentration, while the degree of graphitization is increased [64,144–146]. In contrast, gaseous decomposition products, such as hydrogen or methane, should increase with higher fluences as the decomposition to smaller molecules is favored in comparison to long-chained hydrocarbon formation.

**Figure 13:**
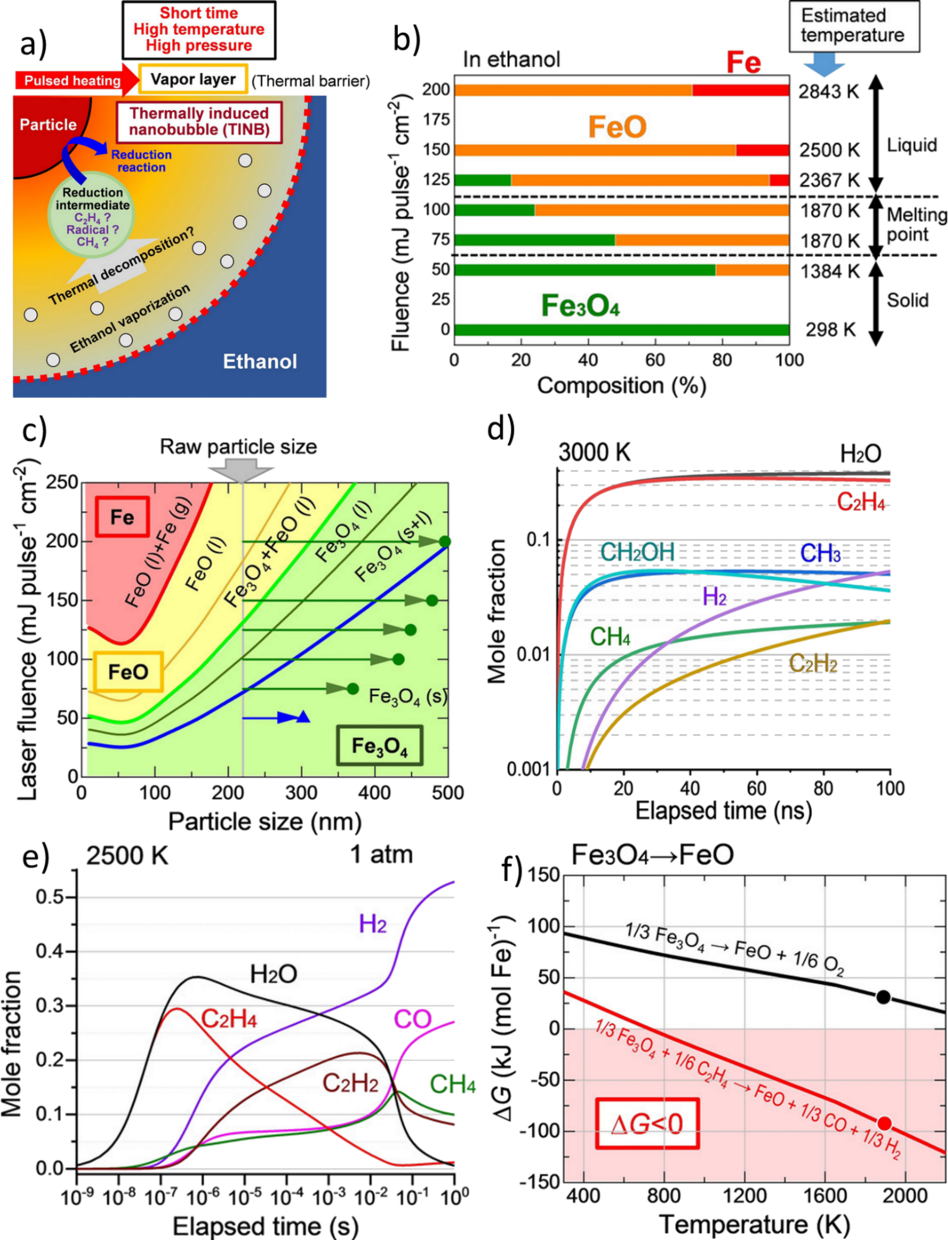
(a) Schematic illustration of the laser melting of a particle in ethanol and the formed nanobubble at the interface. (b) Product compositions of laser-irradiated particles and their estimated attained temperatures during the melting process. (c) Size–fluence diagram calculated for Fe_3_O_4_ during laser irradiation and the stable phases. Blue triangle and green circles are experimental data of non-spherical and spherical particles, respectively. (d) Mole fractions of generated components by thermal decomposition of ethanol at a temperature of 3000 K. (e) Long-time mole fraction evolutions of generated decomposition products of ethanol at a temperature of 2500 K. (f) Temperature dependence of the free Gibbs enthalpy for the reduction of Fe_3_O_4_ by thermal decomposition (black line) and ethylene-mediated thermal decomposition (black line). The melting point of Fe_3_O_4_ is shown as red and black dots. Figure 13 was republished with permission of Wiley, from [171] (“Reduction Mechanism of Transition Metal Oxide Particles in Thermally Induced Nanobubbles during Pulsed Laser Melting in Ethanol” by K. Suehara et al., *ChemPhysChem*, vol. 22, issue 7, © 2021 Wiley-VCH GmbH); permission conveyed through Copyright Clearance Center, Inc. This content is not subject to CC BY 4.0.

This fluence-dependent behavior was recently discussed for the laser melting in liquids, which was used to alter the size and form of nanoparticles, for example, for α-Fe_2_O_3_ in ethanol and ethyl acetate by Shakeri et al. [125] and various oxides in ethanol by Suehara and coworkers [171]. While Shakeri et al. performed mass spectrometry experiments and focused their calculations on the found products (ethyl aldehyde, ethanol, and butane), Suehara et al. directed their calculations to the reactive intermediates that cannot be detected ex situ. They proposed ethanol vaporization during the melting process followed by thermal decomposition to reductive intermediates such as radicals, methane, and ethylene during thermally induced bubble formation, which is shown in Figure 13a. They further estimated the attained temperatures during the melting process for the used fluences (Figure 13b) as well as stable phases in a size–fluence diagram (Figure 13c). Normally, Fe_3_O_4_ should decompose to FeO at around 2500 K, but this reaction step has already been found for temperatures of 1400 K, which is before particle melting occurs. This supports the hypothesis of formed reductive intermediates. Since the particle size increases during the melting process, the particle temperature was reported to be close to the melting point of Fe_3_O_4_. Thus, the particles were likely not reduced to FeO or Fe by thermal decomposition. Hence, Suehara et al. performed computational simulations of the first 100 ns of melting (Figure 12d) as well as for the long-time evolution (Figure 13e) and reported the formation of various species (C_2_H_4_, CH_2_OH, CH_3_, CH_4_, C_2_H_2_, H_2_, and H_2_O) by thermal decomposition. They concluded that C_2_H_4_ was the main species involved in the reduction of the metal oxides, as the C_2_H_4_-mediated reaction was calculated to have a negative free Gibbs enthalpy (Figure 13f). They further calculated decomposition pathways for acetone and found CH_3_ radicals to be the reducing species, while the reduction reaction was hampered in hexane because of particle aggregation and rapid sedimentation. Overall, the authors concluded that the reaction products are determined by thermodynamic relations, while there were less kinetic effects during the irradiation [171].

As the results from Suehara et al. suggest a thermodynamic connection to the formed reaction products, a mathematical approach to correlate the attained temperatures during the nanoparticle synthesis process with the decomposition reactions and possibly formed intermediate products is needed. However, because of the abundance of possibilities during the pyrolysis steps, further research with a focus on the analysis of liquid by-products during the processes is of utmost importance to narrow down the range of potentially formed products. This also needs to be assessed critically in terms of the often-staged “ligand-free” or “purity” claim of LSPC, as such non-volatile by-products during laser synthesis in organic liquids are likely to adsorb on the produced colloidal nanoparticle’s surface.

**Figure 14:**
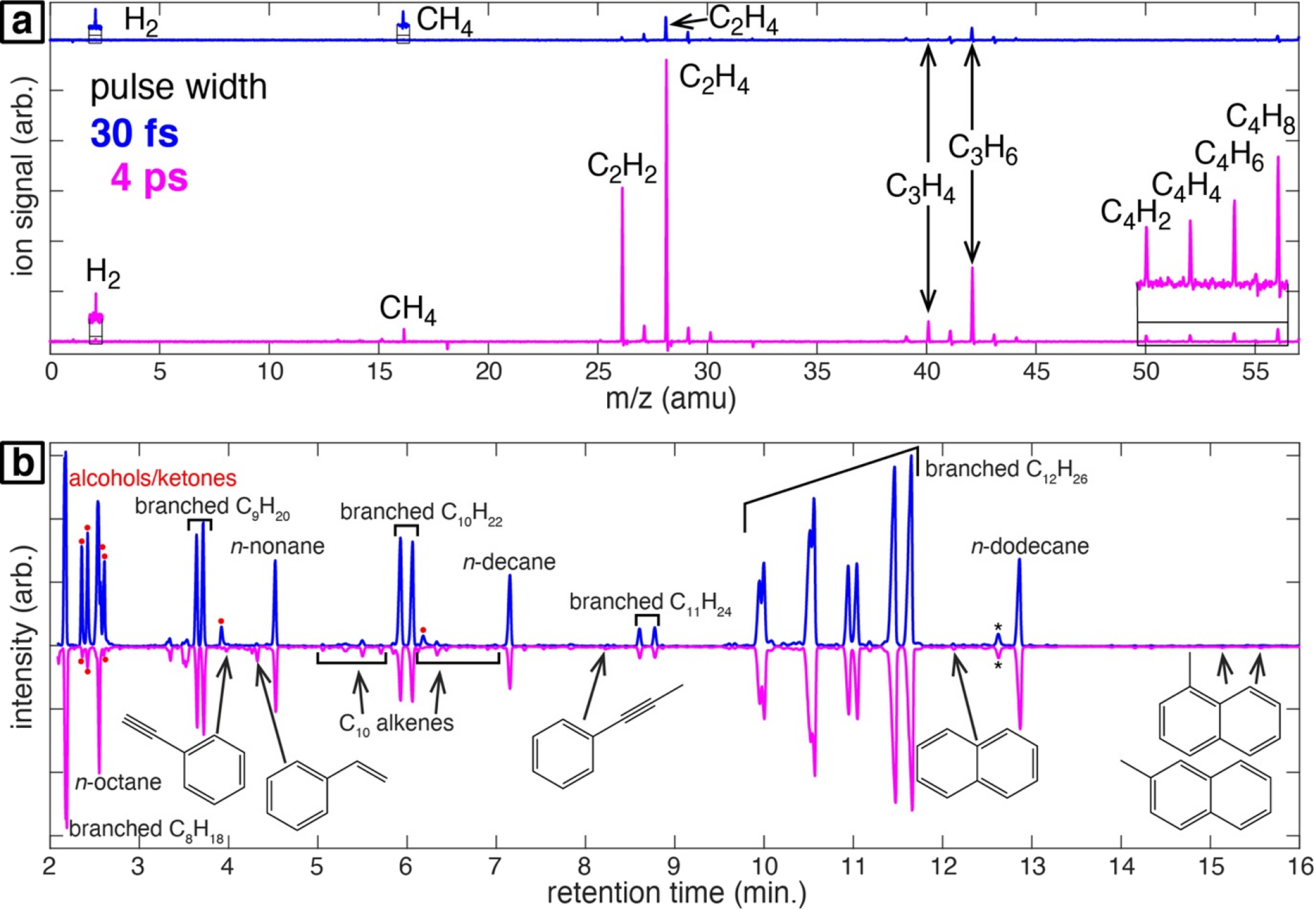
Comparison of irradiation of *n*-hexane with laser pulses with widths of 30 fs (blue) and 4 ps (magenta). (a) Laser mass spectra showing detected gas products. (b) Gas chromatograms with indicated products at different retention times. Figure 14 was adapted with permission from [117]. Copyright 2023 American Chemical Society. This content is not subject to CC BY 4.0.

Suehara et al. and Shakeri et al. investigated the thermodynamic influence on the solvent’s decomposition. However, literature on laser chemistry often reports the use of few-picosecond and femtosecond lasers [52,115,117–121,172]. These ultrashort pulses induce a stronger interaction of the laser irradiation with the solvent and, hence, a higher fraction of by-products. Frias Batista et al. recently investigated the differences in by-product formation during ns- and fs-LRL of [AuCl_4_]^−^ and found results in line with the proposed stronger interaction. The mass spectra of fs-LRL in isopropyl alcohol not only showed a larger variety of by-products but also that the formation rate of, for example, acetone was higher than that for ns-LRL (see Figure 6) [121]. The same authors also found that few-picosecond pulses induce even more rapid formation of by-products than femtosecond pulses during the irradiation of organic solvents [117]. For instance, irradiation of *n*-hexane with 4 ps pulses results in a one order of magnitude increase in gas yields measured by mass spectrometry (Figure 14a). Moreover, irradiation with 30 fs pulses resulted in higher yields of aliphatic alcohols and ketones, due to oxidation by dissolved O_2_. Irradiation with 4 ps pulses resulted in small yields of aromatic products including phenylacetylene, styrene, and naphthalenes (Figure 14b). These results were rationalized by the dominance of avalanche ionization processes for few-picosecond pulsed irradiation, which produces electrons with higher kinetic energy and results in a more violent collapse of cavitation bubbles.

Another factor for solvent chemistry is the applied laser wavelength. While the initiation of chemical reactions by irradiation with wavelengths resonant with an electronic transition is established in organic chemistry, the influence of the wavelength on laser-induced reactions is rarely discussed. In general, organic solvents tend to absorb light in the UV range up to 300 nm such that lower wavelengths are not feasible. As such, the reaction rate during LSPC should be higher when deep UV wavelengths, such as the fourth harmonics of solid-state lasers or excimer lasers, are used compared to IR irradiation. While the influence of the wavelength on the formation of graphene nanosheets by LAL [144], LFL of Au nanoparticles [25], and LRL of AuCl_3_ [173] were already reported, a comparison of chemical reaction rates during LSPC in organic solvents is lacking.

## Summary and Conclusion

To control nanoparticle properties such as surface oxidation, doping, or carbon shell formation during LSPC, it is crucial to understand the chemical reactions between the liquid and the forming nanoparticles during the different LSPC method variants. To date, the majority of studies investigating the chemistry of laser-induced reactions during LSPC have focused on aqueous media. Hydrogen, oxygen, and hydrogen peroxide can be detected during LSPC in water. The associated redox reactions involve a complex network of radical-related reaction kinetics and the release and subsequent capture of free electrons. Accordingly, oxides and hydroxides on the surface of nanoparticles have been evidenced whereby their quantity depends on the standard electrochemical reduction potential of the nanoparticle element. Because of the consumption of oxygen during the oxidation, which is particularly vigorous when ablating and/or laser processing elements with a low standard electrochemical reduction potential, the hydrogen content of the permanent gases released increases and may deviate from the stoichiometric ratio of 1:2 (O_2_/H_2_). Additionally, the formation of H_2_O_2_ has also been reported and discussed in terms of additional catalytic reactions triggered by the nanoparticle itself (i.e., when ablating platinum in water). It can be concluded that the laser-induced reactions between the laser-ablated material and water as well as the related radical kinetics are fairly well described to date, at least at a technologically feasible level. However, particularly during the last decade, many studies, especially those involving oxidation-sensitive elements (or compounds) are conducted in organic solvents to suppress their oxidation, where the solvent acts as a sacrificial reduction agent. However, in this scenario, the fundamental reactions and solvent decomposition pathways are much less well understood than in water.

A common observation for LSPC of metals in organic liquid is the formation of carbon-containing nanoparticles, where the carbon species are either located in the surface-near regions of the metal nanoparticles (carbon shell) or throughout the whole nanoparticle volume (carbon-doped or crystalline carbides) [101]. It has been inferred that the used solvent influences the crystallinity of the carbonaceous species [100], the chemical structure of the metal core [100,156,174] of the carbon shell [104–105], as well as the overall degree of metal oxidation [100]. The metal core’s composition is also influenced by atmospheric conditions [86,97–98], resulting in another parameter that affects the carbon formation process that remains to be elucidated. Gaseous and short-chained liquid hydrocarbons can be found during LSPC in organic solvents, besides hydrogen, CO, and CO_2_. Here, the laser-induced bond cleavage of the respective solvents leads to the formation of smaller fragments, which may result in permanent gases. The formation of hydrogen can often be attributed to a complete conversion of the hydrocarbons to elemental carbon. The bond dissociation energies are lower for organic liquids than for water, such that the cleavage into smaller molecules requires less energy than the splitting of water. The gas formation rates have been observed to be in linear correlation to the enthalpy of vaporization of the solvent [107]. When comparing different laser pulse durations, the current literature indicates that nanosecond laser pulses form fewer olefinic and more carbonaceous products, while experimental results using femtosecond and picosecond laser pulses appear to enhance coupling reactions and the formation of dimers. The still rather complex and scattered data basis is potentially linked to the fact that the laser irradiation can lead to both the formation of radicals and general pyrolysis, depending on the employed laser pulse duration, fluence, as well as effects of metal-catalyzed reactions, particularly when transient metal nanoparticles are laser-synthesized or -processed. However, the formation of longer-chained aliphatic and olefinic liquid by-products remains elusive and requires significantly more attention in future studies on LSPC in organic solvents.

As for the nanoparticles that are obtained from LSPC in organic solvents, significantly more literature data can be found in particular for the LAL of metals in solvents, including several hypotheses addressing the formation of metal–carbon core–shell nanoparticles. While one hypothesis assumes that carbon atoms from the decomposing solvent diffuse into the superheated liquid-like metal and segregate to the surface during cooling, the other two hypotheses assume that the metal nanoparticles form more or less independently, while the carbon species enrich on the surface directly from the liquid or in the vapor phase during the formation of the cavitation bubble. Overall, the current literature indicates that the reactivity/solubility of carbon with/in the respective nanoparticle material is crucial, particularly in cases where metal carbide nanoparticles, metallic glass nanoparticles [23–24], MOFs [175–176], or polyoxometalates [177–178] were obtained directly from the ablation of metal targets in organic solvents. Yet, to gain better control, and even more importantly, a predictive tool for the nanostructures and compositions that form in the respective organic liquid, a more comprehensive mechanistic understanding that summarizes the reactions occurring during the laser-based synthesis is needed. Currently, this aspect remains a black box demanding a more integrated collaboration between experimental and theory groups to conduct mechanistic and empirical studies to establish correlations between the organic solvents used and, for example, gas formation rate, carbon layer thickness, chemical nature of the carbon layer, and molecular decomposition products, with the help of computational methods to elucidate reaction and particle formation kinetics. In addition, time-resolved techniques as used in previous studies, for example, to analyze the temporal evolution of the nanoparticle size distribution [179], elemental composition [180], and the fragmentation mechanisms itself [72], will help to link computational investigations with advanced (high-resolution) empirical studies of the final nanostructure and solvent composition. The rapid progress in both fields of mechanistic time-resolved pump–probe studies and coarse-grained molecular dynamics simulations of the laser ablation and fragmentation processes provides an optimistic perspective on this matter. As with practically all topics in modern sciences, only a collaborative and holistic effort of the pulsed laser application and materials science communities will raise us to the next level of understanding, which is needed for a less empirical and more knowledge-driven materials design when using organic solvents as liquids during LSPC.

While the aforementioned directions and challenges have focused on unravelling a mechanistic understanding of the LSPC process, more research is also needed to advance LSPC in organic liquids toward the practical use in industrial processes regarding biomedicine and catalysis. For biomedicine, the employment of organic liquids is required to avoid oxidation or to create defined nanoparticle compositions and structures, such as magneto-plasmonic FeAu or Fe@Au [181–183] or boron [184–185] nanoparticles. In catalysis, the protection of the active sites against oxidation or nanoparticle surface segregation requires organic liquids as well. Here, liquids such as propylene carbonate [186], ethanol [125–126], or acetone [86,97–98] are required. An emerging field in electrocatalysis is the synthesis of high-entropy alloy (HEA) nanoparticles or compositionally complex alloy nanoparticles (for nomenclature, refer to [187]). Alternative (wet chemical or gas-phase) HEA nanoparticle synthesis methods either require elevated temperatures [188] or conductive substrates [189], and substrate loading cannot be adjusted independent from particle size [190]. In contrast, room-temperature LSPC gives access to quinary [191–192] or senary [23] HEA colloidal nanoparticles independent of a support material. Trying to minimize the employment of precious metals or critical elements, the Cantor HEA and its derivatives have been synthesized by LAL in ethanol or acetonitrile [23,191–192] and LRL in water [34,54,193–195]. These laser-generated HEA nanoscale catalysts showed good electrocatalytical performance in hydrogen evolution reaction (HER) [34,193], oxygen evolution reaction (OER) [34,195], and C–H activation [194]. Moreover, huge quantities are demanded already for throughput screening of alloy nanoparticle series [196], at the gram-to-kilogram scale of supported catalyst, which equals hundreds of milligrams to grams of nanoparticles per sample [197], depending on catalyst loading. Although there is work on high-productivity setups [5,70,198], these setups work with water. But typical organic liquids have lower vapor pressure, lower heat capacity, and higher viscosity, attenuating productivity by larger cavitation bubbles that stick in ellipsoidal shape on the target surface even after collapse [199], persistent microbubbles, and gaseous by-products that shield the laser beam. Moreover, the gaseous side products may pose the risk of inflammation and require special consideration regarding workplace safety. This also includes the consideration of the generated by-products such as hydrogen, methane, and other hydrocarbons stemming from solvent decomposition reactions.

In addition, the laser-based synthesis of nanoparticles in organic liquids is mostly performed to obtain metal, alloy, or carbide/carbon-coated nanoparticles, while sulfides, phosphides, selenides, and so on are significantly less studied, although the process would allow for the use of a wide range of materials, where the main group element may stem from the target and/or the heteroatom of the liquid. In this regard, the focus should be set on nanoparticle materials and structures that are otherwise only accessible through more resource demanding synthesis methods. This applies not only to composition but also to structure as it has been shown that the kinetic contribution of LSPC, in particular, the high cooling rates (or higher undercooling before freezing), gives access to defect-rich particles, beneficial for application in catalysis. The abovementioned aspects of LSPC in organic liquids are only a fraction of the open questions in this field, but they show that further intensive research is needed to realize the potential of LSPC, as the laser-generated materials have unique properties that can be relevant for different applications.

## Data Availability

Data sharing is not applicable as no new data was generated or analyzed in this study.
